# Drivers underpinning the malignant transformation of giant cell tumour of bone

**DOI:** 10.1002/path.5537

**Published:** 2020-10-06

**Authors:** Matthew W Fittall, Iben Lyskjær, Peter Ellery, Patrick Lombard, Jannat Ijaz, Anna‐Christina Strobl, Dahmane Oukrif, Maxime Tarabichi, Martin Sill, Christian Koelsche, Gunhild Mechtersheimer, Jonas Demeulemeester, Roberto Tirabosco, Fernanda Amary, Peter J Campbell, Stefan M Pfister, David TW Jones, Nischalan Pillay, Peter Van Loo, Sam Behjati, Adrienne M Flanagan

**Affiliations:** ^1^ The Francis Crick Institute London UK; ^2^ Department of Pathology (research) University College London Cancer Institute London UK; ^3^ Cancer, Ageing and Somatic Mutation Wellcome Trust Sanger Institute Hinxton UK; ^4^ Department of Molecular Medicine Aarhus Universitet Aarhus Denmark; ^5^ Department of Cellular Pathology University College London NHS Trust London UK; ^6^ Department of Histopathology Royal National Orthopaedic Hospital NHS Trust Stanmore UK; ^7^ Hopp Children's Cancer Center Heidelberg (KiTZ) Heidelberg Germany; ^8^ Division of Pediatric Neurooncology, German Cancer Research Center (DKFZ) and German Cancer Consortium (DKTK) Heidelberg Germany; ^9^ Institute of Pathology University Hospital Heidelberg Heidelberg Germany; ^10^ Department of Human Genetics University of Leuven Leuven Belgium; ^11^ Department of Pediatric Hematology and Oncology University Hospital Heidelberg Heidelberg Germany; ^12^ Pediatric Glioma Research Group German Cancer Consortium (DKTK), German Cancer Research Center (DKFZ) Heidelberg Germany; ^13^ Department of Paediatrics University of Cambridge Cambridge UK

**Keywords:** bone, sarcoma, giant cell tumour, genomics, methylation, osteoclast, osteoblast, histone, cyclin D1, epigenetic, drivers, transformation

## Abstract

The rare benign giant cell tumour of bone (GCTB) is defined by an almost unique mutation in the H3.3 family of histone genes *H3‐3A* or *H3‐3B*; however, the same mutation is occasionally found in primary malignant bone tumours which share many features with the benign variant. Moreover, lung metastases can occur despite the absence of malignant histological features in either the primary or metastatic lesions. Herein we investigated the genetic events of 17 GCTBs including benign and malignant variants and the methylation profiles of 122 bone tumour samples including GCTBs. Benign GCTBs possessed few somatic alterations and no other known drivers besides the H3.3 mutation, whereas all malignant tumours harboured at least one additional driver mutation and exhibited genomic features resembling osteosarcomas, including high mutational burden, additional driver event(s), and a high degree of aneuploidy. The H3.3 mutation was found to predate the development of aneuploidy. In contrast to osteosarcomas, malignant H3.3‐mutated tumours were enriched for a variety of alterations involving *TERT*, other than amplification, suggesting telomere dysfunction in the transformation of benign to malignant GCTB. DNA sequencing of the benign metastasising GCTB revealed no additional driver alterations; polyclonal seeding in the lung was identified, implying that the metastatic lesions represent an embolic event. Unsupervised clustering of DNA methylation profiles revealed that malignant H3.3‐mutated tumours are distinct from their benign counterpart, and other bone tumours. Differential methylation analysis identified *CCND1*, encoding cyclin D1, as a plausible cancer driver gene in these tumours because hypermethylation of the *CCND1* promoter was specific for GCTBs. We report here the genomic and methylation patterns underlying the rare clinical phenomena of benign metastasising and malignant transformation of GCTB and show how the combination of genomic and epigenomic findings could potentially distinguish benign from malignant GCTBs, thereby predicting aggressive behaviour in challenging diagnostic cases. © 2020 The Authors. *The Journal of Pathology* published by John Wiley & Sons, Ltd. on behalf of The Pathological Society of Great Britain and Ireland.

## Introduction

Giant cell tumour of bone (GCTB) is a locally destructive benign tumour, prone to local recurrence. This tumour presents predominantly at the site of the mature epiphysis/epimetaphysis of the long bones, particularly the distal femur and proximal tibia in the third and fourth decades of life [1]. GCTB is defined by a near universal (96%) pathognomonic *H3‐3A*:p.G34W missense mutation [2, 3, 4, 5, 6]. Two unexplained phenomena in GCTB are of particular interest, firstly that lung metastases occur despite the absence of malignant histological features in either the primary or the metastatic lesions [7] and, secondly, that the characteristic *H3‐3A* mutation is occasionally found in primary malignant bone tumours which often share features with conventional GCTB [4, 8]. We set out to explore the genomic events underlying these phenomena using whole genome sequencing and genome‐wide methylation profiling using methylation array and whole genome bisulphite sequencing.

## Materials and methods

### Patient samples

Patients provided their written and informed consent to provide samples for this study via the UCL Biobank for Health and Disease, based at the Royal National Orthopaedic Hospital. This was approved by the National Research Ethics Service (NRES) Committee Yorkshire & The Humber – Leeds East (15/YH/0311). DNA was extracted from areas of fresh frozen tissue selected by bone pathologists (AMF/RT/FA/PE). All samples were selected after immunostaining for the mutated H3.3 G34 protein as previously described [4]. Matched normal DNA was acquired from blood samples.

### DNA extraction

Fresh frozen tumour samples were embedded in Tissue‐Tek® O.C.T.™ (Tissue‐Tek, Sakura Finetek Europe BV, Alphen aan den Rijn, The Netherlands) and sectioned at 3–5 μm thickness using a cryostat. Haematoxylin and eosin (H&E)‐stained sections were reviewed for tumour type and uniformity, to ensure a tumour content of greater than 50%. DNA was extracted using an automated magnetic bead extraction and purification system following the manufacturer's protocols (Prepito DNA Tissue10 Kit; Perkin Elmer Ltd, Bucks, UK). Matched normal DNA was acquired from blood using a column‐based system (QIAamp DNA Blood Maxi kit; Qiagen, Manchester, UK). DNA concentration was assessed using a fluorometric assay (Picogreen; Thermo Fisher Scientific, Paisley, UK) and quality using a PCR assay followed by gel electrophoresis.

Only DNA that was of suitable concentration (1 μg total DNA) was used for whole genome sequencing.

### SNP and methylation arrays

SNP analysis was performed on 200 ng of high‐quality genomic DNA using the Illumina Human Infinium Omni2.5‐8 beadchip (Illumina Inc, San Diego, CA, USA) following the Infinium LCG Assay (15023141_A, June 2010) protocol. DNA methylation analysis was performed on 500 ng of high‐quality bisulphite‐converted genomic DNA using either Illumina Infinium Human Methylation 450 or Methylation EPIC 850 k arrays. Sample processing was carried out in accordance with the Infinium HD Assay (15019519_B, 2011 or 15019519 v01) protocol. All beadchips were scanned using the iScan scanner with autoloader. Raw data were quality controlled and converted into normalised LogR and B‐Allele Frequency tracks using Illumina Genome Studio (2.0.4). Pre‐processing and quality control were performed. Details are provided in supplementary material, Supplementary materials and methods.

### Array analysis

SNP array copy number profiles were produced using ASCAT (v2.5.1). Methylation array‐based copy number profiles were generated using the *conumee* package (v1.18.0) and a bespoke adaptation of the principles utilised by ASCAT (see supplementary material, Supplementary materials and methods). Unsupervised clustering was performed by hierarchical clustering using the 5000 most variable probes across samples after scrutiny for batch effects (see supplementary material, Supplementary materials and methods). Differentially methylated probes and regions were detected using the ChAMP package (v2.14.0). Gene set enrichment analysis (GSEA) was performed using an adapted approach from the ebBayes function in the ChAMP package [9]. Bespoke analysis for regional differences in methylation was performed using Circular Binary Segmentation (CBS) functions from the DNACopy package (v1.58.0) based on a signal–noise ratio for each methylation probe (see supplementary material, Supplementary materials and methods).

### Whole genome bisulphite sequencing

Whole genome bisulphite sequencing was performed on seven of the samples: PD30981a, PD30982a, PD30984a, PD30985a, PD3788d, PD3795d, and PD4915d. Oxidative bisulphite conversion and library preparation were done using the Cambridge Epigenetix Truemethyl Whole Genome kit following the manufacturer's instructions (CEGX, Cambridge, UK; TrueMethyl® Whole Genome Kit User Guide). The efficiency of bisulphite treatment was determined using control probes; 90.6% of 5‐methylcytosine remained unconverted and 100% of unmethylated cytosines were converted into thymines. Hydroxymethylation was not detected using this kit as 91.7% of 5‐hydroxymethyl cytosine was converted into thymine. Libraries were sequenced using an lllumina HiSeqX (Illumina Cambridge, Ltd, Little Chesterford, UK) using a 150 bp paired‐end run. Both mapping of reads to GRCh37 and methylation calling were done using Bismark (https://github.com/FelixKrueger/Bismark).

### Whole genome sequencing

For whole genome sequencing, the Illumina no‐PCR library protocol was used to construct short insert 500 bp libraries, prepare flowcells, and generate clusters. Whole genome sequencing was performed using the Illumina HiSeq 2000 or 2500 platform, using 100 bp paired‐end reads. Samples PD37332, PD3788, PD3795, PD38328, PD38329, PD4915, and PD4922 were sequenced using the HiSeq XTen platform using 150 bp paired‐end libraries.

### Variant detection, validation, and analysis

The Cancer Genome Project (Wellcome Trust Sanger Institute) variant calling pipeline was used to call somatic mutations (versions as below). All variant calling algorithms were used with standard settings with limited post‐processing filtering, and variants were analysed using a previously documented strategy [10] (see supplementary material, Supplementary materials and methods). Variants were considered as potential drivers if they presented in established cancer genes (COSMIC v85 [11]). Tumour suppressor coding variants were considered if they were annotated as functionally deleterious by the VAGrENT algorithm (http://cancerit.github.io/VAGrENT/). Disruptive rearrangements or homozygous deletions of tumour suppressors were also considered. Additionally, homozygous deletions were required to be focal (<1 Mb in size). Mutations in oncogenes were considered to be driver events if they were located at previously reported hot spots (point mutations) or amplified the intact gene. Amplifications also had to be focal (<1 Mb) and result in at least five copies in diploid genomes, or four copies more than the modal major copy number in genome‐duplicated samples.

### Copy number scoring

A sample was considered whole genome duplicated (WGD) when the modal total copy number was greater than 2. The baseline total copy number was considered as 4 for WGD samples and 2 for others. Autosomal copy number segments were then scored as the difference from this baseline: no difference (0), total copy number of 0 (homozygous deletion, 2), total copy number ≥ 3 + baseline (amplification, 2), other score not equal to baseline (1). Scores were normalised relative to the length of the chromosome, summed, and then divided by the theoretical maximum (44). Aneuploidy was validated using image cytometry on nuclei extracted from formalin‐fixed tissue sections (see supplementary material, Supplementary materials and methods).

### Mutation clustering, phylogenetic reconstruction, and timing analyses

The algorithm DPClust (v2.2.6) and its pre‐processing pipeline (v1.0.8) were used to cluster mutations according to the fraction of cancer cells (cancer cell fraction, CCF) in which they were found, as described previously [12] (see supplementary material, Supplementary materials and methods). Phylogenetic reconstruction was performed using the pigeon‐hole principle [12]. In brief, subclones were designated to be nested within a clone or another subclone if their combined CCF exceeded that of their parent.

Initial timing analysis required the transformation of individual mutation allele frequencies into mutation copy number. This was performed using the equation:

$$\mathrm{MCN}=\frac{\mathrm{VAF}\left(\rho \times \mathrm{TCN}+2\left(1-\rho \right)\right)}{\rho }$$

where *MCN* is the mutation copy number, *ρ* is the sample purity, and *TCN* is the local total tumour copy number.

For WGD timing, deamination (clock‐like, C>T mutations at CpG dinucelotides) mutations were selected from regions of balanced gain (2 + 2) or LOH (2 + 0). A probabilistic approach to WGD timing was taken with confidence intervals generated by bootstrapping the underlying mutations (see supplementary material, Supplementary materials and methods).

### Data analysis

General data analysis was performed in R (3.5.3 and 3.6.0) in RStudio (1.1.383), with bespoke scripts.

## Results and discussion

We started our investigation by performing whole genome sequencing (WGS) on seven primary malignant bone tumours possessing an *H3‐3A/B*:p.G34W/R mutation, one case of metastatic GCTB, and nine conventional GCTBs also harbouring an *H3‐3A* mutation for which we had frozen tissue with corresponding germline DNA from blood samples (supplementary material, Table S1 **)**. We used the analysis pipeline of the Cancer Genome Project to generate catalogues of somatic mutations, indels, structural variants, and copy number changes as outlined in the Materials and methods section and supplementary material, Supplementary materials and methods, and a previously reported strategy to identify putative drivers [10].

**Figure 1:**
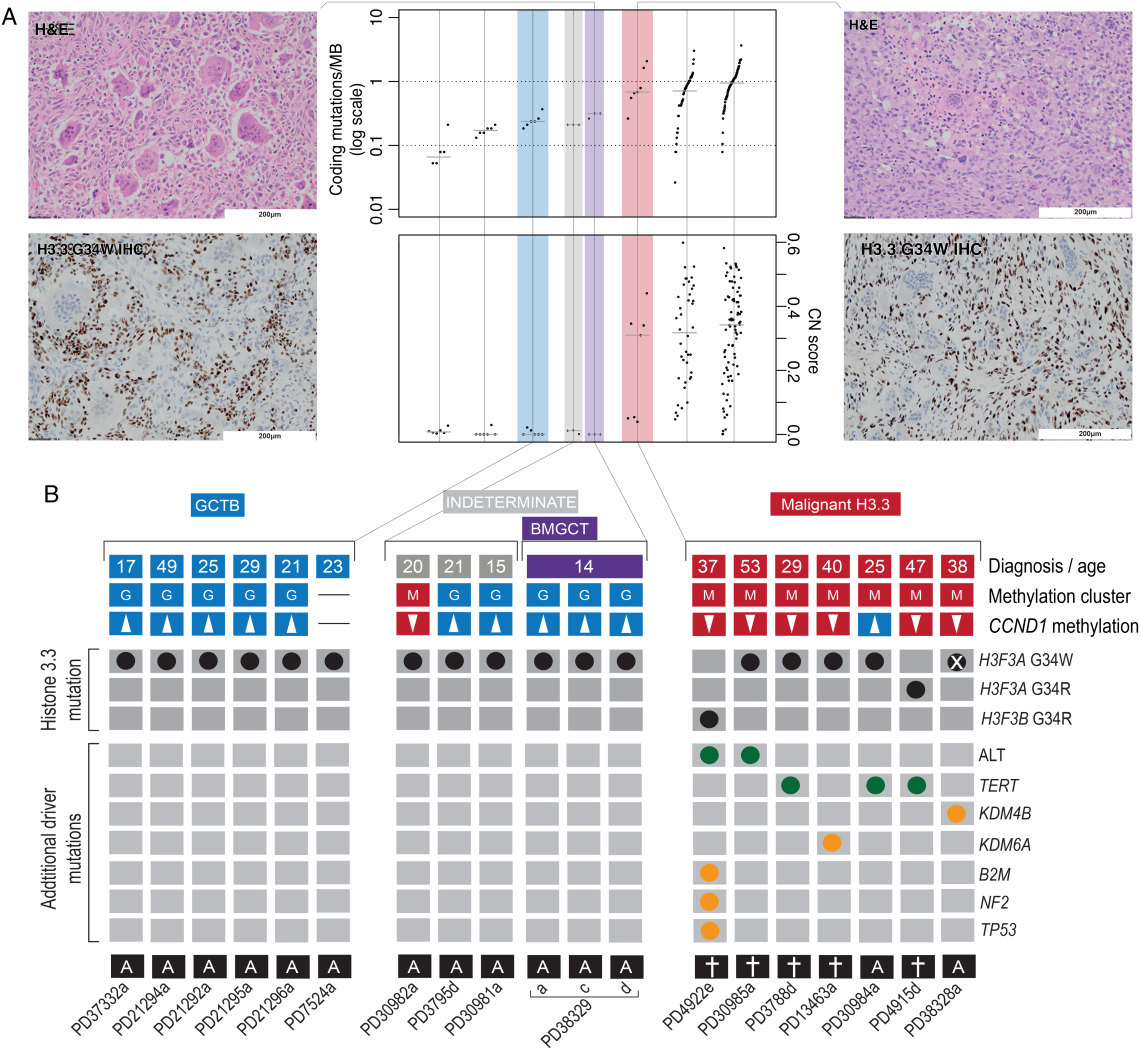
Landscape of H3.3‐mutant tumours. (A) Photomicrographs of H&E and H3.3 G34W immunostained tissue sections of a benign metastasising (far left) and malignant giant cell tumour of bone (far right). Both tumours are osteoclast‐rich but the malignant neoplasm exhibits cellular atypia. Mutational burden of samples in comparison with selected other mesenchymal tumours (centre panel): osteoblastoma [20], chondroblastoma [5], chondrosarcoma [21] (*exome data only; SVs not shown), and osteosarcoma [10]. (B) The genomic and methylation classification of sequenced tumours. From top to bottom: clinical diagnoses and age, unsupervised methylation cluster assignment, *CCND1* promoter methylation status (hypermethylation defined as a mean *CCND1* promoter methylation beta value greater than the fifth centile for GCT), and a tileplot of curated drivers; clinical outcomes are shown underneath (more detailed clinical outcomes are shown in supplementary material, Table S1). Note sample PD38328a had undergone deletion of the *H3‐3A* locus, which had been present on the pre‐resection biopsy (supplementary material, Figure S11). Sample PD37332a was a biphasic tumour with one component showing classical features of a benign GCTB merging with a low‐grade osteosarcomatous component; so, although classified as benign (and the methylation array was from the benign component), the tumour overall would be considered a malignant GCTB.

Benign GCTBs genomically resemble other benign mesenchymal tumours (Figure 1A); they possess few somatic changes of any type and no known plausible driver mutations other than the canonical *H3‐3A* mutation (medians: 640 substitutions, 43 indels, 7 structural variants; Figure 1A and supplementary material, Figure S1). In contrast, we found that histologically malignant bone tumours with a p.G34 H3.3 mutation possess genomic features resembling osteosarcoma. They revealed an increased burden of somatic variants, and broadly divided into two groups: 3/7 tumours exhibited a modest increase in mutations (medians: 1815 substitutions, 86 indels, 21 structural variants) and the remaining four possessed a greater mutation burden (medians: 4177 substitutions, 205 indels, 108 structural variants; Figure 1A and supplementary material, Figure S1).

Unlike osteosarcoma, malignant H3.3‐mutated bone tumours are enriched with mutations suggesting telomere dysfunction. Two tumours had mono‐allelic G>A mutations 124 bp upstream of the *TERT* transcription start site, reported to increase promoter binding [13]. Another sample, PD3788d, had a complex rearrangement event, resembling chromothripsis, encompassing *TERT*, resulting in the juxtaposition of the gene *MEGF10* and the *TERT* promoter (supplementary material, Figure S2). *MEGF10* is reported to be under the control of a super‐enhancer in the dbSUPER database [14]. Two other malignant samples, PD4922e and PD30985a, were identified as having markedly elongated telomeres (Figure 1B and supplementary material, Figure S1), a finding consistent with alternative lengthening of telomeres (ALT) pathway activation, which is usually mutually exclusive with *TERT* alteration [15, 16]. In keeping with this pattern of ALT, recently reported in other sarcoma types [17], these tumours possessed highly rearranged genomes, the telomeres of which comprised conventional (‘TTAGGG’) repeats and had undergone loss of heterozygosity at the *RB1* locus (supplementary material, Figure S3). In total, 5/7 malignant tumours had evidence of a *TERT*‐mutated phenotype. In contrast, only *TERT* amplifications have previously been reported in osteosarcoma [10]. The remaining two malignant tumours both harboured biallelic losses of an additional histone lysine demethylase, *KDM4B* or *KDM5A* (supplementary material, Figure S3). All malignant tumours had therefore acquired at least one additional driver mutation in addition to the G34W.

The degree of aneuploidy observed in 3/7 malignant tumours, against a backdrop of almost ubiquitously diploid GCTBs (supplementary material, Figure S4), allowed the ordering and timing, in real time, of the most significant mutational events. In all three cases (PD4922e, PD30985a, and PD3788d), whole genome duplication (WGD) had occurred in adulthood, but several years prior to diagnosis. Chromothripsis had occurred subsequent to this. In 2/3 samples (PD4922e, PD30985a) with informative data, the *H3‐3A/B* mutation had also been duplicated, demonstrating its occurrence as an earlier mutational event prior to WGD (supplementary material, Figure S5). This is consistent with the progression of these malignant tumours from GCTBs.

We next investigated the ‘benign metastasising GCTB’ [1]. In contrast to malignant tumours, the morphology of both the metastases and the primary lesion (PD38329a/c/d) was that of a conventional GCTB, which was reflected in the low mutational burden and the absence of additional driver mutations (Figure 1B and supplementary material, Figure S1). Leveraging the independent sampling across these three tumour samples increased the power to define the clonality of mutations. Clonal mutations are those found in all tumour cells, whereas those in only a fraction of cells are considered subclonal. The primary tumour (PD38329a) and the two metastases (PD38329c and PD38329d) each possessed a group of private mutations, present in only that tumour sample. Furthermore, one cluster of mutations (supplementary material, Figure S6) was found to be common but subclonal in all samples. This suggests that both metastases were seeded by at least one cell possessing those mutations and at least one cell that did not, a process known as polyclonal seeding.

**Figure 2:**
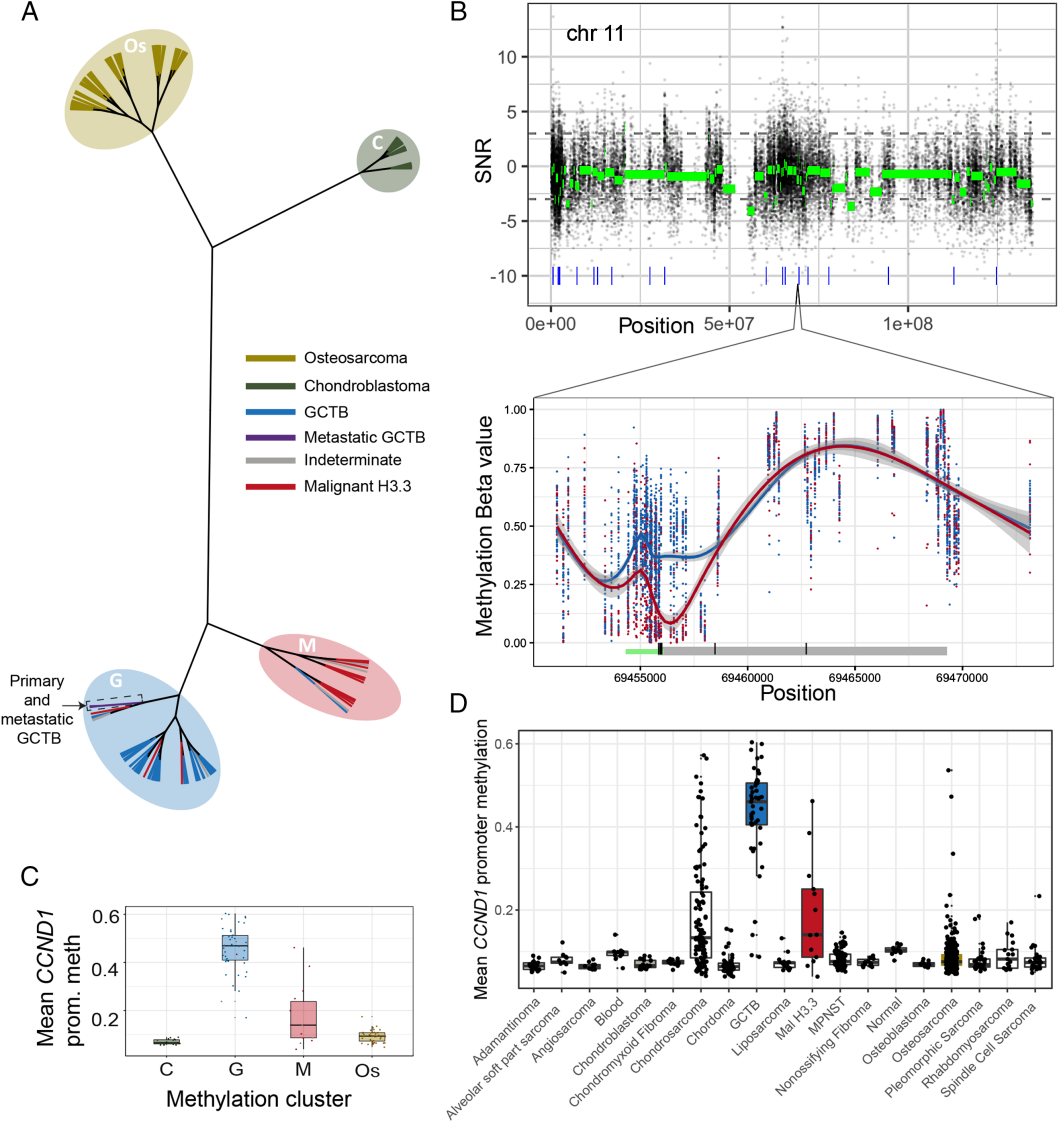
Methylation changes of H3.3‐mutant tumours. (A) Hierarchical (unrooted) clustering of tumours. Leaves are coloured by diagnosis and the methylation clusters annotated with shaded ovals. (B) Analysis of methylation differences between malignant (‘M’) and benign (‘G’) tumours (*n* = 13 and 42, respectively) across chromosome 11 (upper) and across *CCND1* (lower). Raw (black) and segmented (green) signal–noise ratios (SNR; >0 shows greater methylation in malignant tumours) are plotted. Blue ticks in the upper plot represent DMRs. In the lower plot, raw methylation beta values across *CCND1* are shown for each sample. The underlying schematic represents the *CCND1* gene body (grey) and the predicted promoter (green). (C) Mean *CCND1* promoter methylation across the clustered samples from A and (D) a variety of other tissues.

To explore the epigenetic differences between malignant and benign H3.3‐mutated bone tumours, we collected additional tumours for DNA methylation array analysis. This collection (*n* = 121) included some of the sequenced samples, osteosarcomas without H3.3 mutations, and chondroblastomas, a benign tumour with an alternative H3.3 mutation, *H3‐3B*:p.K27M (supplementary material, Table S2). Unsupervised clustering based on the most variable methylation probes recapitulated the diagnostic groups (Figure 2A and supplementary material, Figure S7). Furthermore, while closely related to conventional GCTB, the malignant H3.3‐mutant tumours formed a distinct clade. The benign metastasising samples clustered with the benign GCTB group.

To unpick the methylation differences underlying the separate clustering of the benign and malignant H3.3‐mutated tumours, differentially methylated regions (DMRs) were identified. This revealed focal changes in a small number of specific gene promoters. Of 74 DMRs identified, 56 were located around gene transcriptions start sites (supplementary material, Figure S8). The most statistically significant DMR was also the only one identified in a plausible known cancer driver gene, *CCND1*, which encodes the cell cycle regulator, cyclin D1. Differential methylation spanned a promoter region of 1500 bp either side of the transcription start site, a finding validated by bisulphite sequencing (supplementary material, Figure S9). Comparing the mean methylation level across this promoter region between different bone and soft tissue tumour types revealed that hypermethylation at this site is specific to GCTB (Figure 2D). Malignant histone‐mutated tumours and chondrosarcomas were the only tumour types with a similar degree of *CCND1* promoter methylation. *CCND1* promoter methylation was concordant with unsupervised methylation clustering groups (Figures 1 and 2). Beyond this, methylation differences were enriched at non‐enhancer intergenic sites; however, those that affected genes did not consistently alter gene pathways. At a broader scale, part of the cluster of histone genes on chromosome 6 was focally hypermethylated in malignant tumours, suggesting additional epigenetic driver events (supplementary material, Figure S10).

Using comprehensive genomic and methylation profiling, we report here the driver events associated with malignant or metastatic progression of GCTBs. Malignant H3.3‐mutated tumours are characterised by a methylation profile that is related to but distinct from conventional GCTB. Histone mutation predates the development of aneuploidy in malignant tumours, which still occurs some years prior to diagnosis. Malignant progression requires additional genetic mutations endowing either telomere or epigenetic dysfunction, and possible additional epigenetic changes altering clusters of histone genes. Once transformed, the histone mutation itself is dispensable to the malignant phenotype (Figure 1 and supplementary material, Figure S11) [18].This combination of genomic and epigenomic findings could potentially distinguish benign from malignant GCTs, thereby predicting aggressive behaviour in challenging diagnostic cases (Figure 1B and supplementary material, Table S3). It also permits malignant GCTB to be classified on a molecular basis distinguishing it from other primary malignant bone tumours. Such a molecular classifier of sarcoma subtypes is under development, despite the challenges generated by their diversity and scarcity. The absence of additional genetic events in metastatic but histologically benign GCTB and the presence of polyclonal seeding support the longstanding hypothesis that they represent an embolic event.

## Author contributions statement

AMF conceived the project. MWF performed the data analyses to which PL, PE, IL, AMF and NP contributed some preliminary analyses. JI performed whole genome bisulphite analysis. A‐CS performed DNA extraction. DO contributed to the image cytometry analysis. AMF, PE, RT and FA curated and reviewed the samples, clinical data, and/or provided clinical expertise. MS, CK, IL and DTWJ contributed to the methylation analyses. MT contributed to the methylation copy number analysis and the timing analyses. JD contributed to discussions. AMF, PVL and SB directed the research. MWF, PVL, SB and AMF wrote the manuscript.

## Supporting information


**Supplementary materials** **and methods**

**Figure S1.** Telomere lengths, mutation burdens, structural variant burdens, and copy number scores for sequenced tumours
**Figure S2.***TERT* rearrangement in PD3788d
**Figure S3.***KDM4B* homozygous deletion and *RB1* loss of heterozygosity
**Figure S4.** SNP and methylation array‐based copy number scores
**Figure S5.** Mutation timing of malignant samples
**Figure S6.** Mutation clustering in metastatic samples
**Figure S7.** Tumour methylation clustering with other malignant bone tumours
**Figure S8.** Selected gene promoters identified as differentially‐methylated regions
**Figure S9.** Whole genome bisulphite sequencing confirmation of *CCND1* differential methylation
**Figure S10.** Differential methylation in the HIST1 cluster
**Figure S11.** The loss of *H3‐3* mutation in the malignant GCTB sample PD38328a

**Table S1.** Clinical characteristics of whole genome sequenced H3.3‐mutant bone tumours
**Table S2.** Additional array samples analysed

**Table S3.** Clinically indeterminate cases

## Data Availability

DKFZ raw methylation array data (IDAT files) were acquired from the authors of Koelsche *et al*, the data having been acquired as described [19]. The authors declare that all data supporting the findings of this study are available within the article and its supplementary files or from the corresponding author on reasonable request. Sequencing data have been deposited at the European Genome‐Phenome Archive (http://www.ebi.ac.uk/ega/) that is hosted by the European Bioinformatics Institute.
