# Supplementary material for: Drivers underpinning the malignant transformation of giant cell tumour of bone
Source: J Pathol. 2020 Oct 6;252(4):433–40. doi: 10.1002/path.5537 (PMC8432151; doi:10.1002/path.5537)
Supplement: Supplementary file 1 — Supplementary materials and methods [file PATH-252-433-s003.docx]

**Drivers underpinning the malignant transformation of giant cell tumour of bone**MW Fittall *et al. J Pathol* DOI: 10.1002/path.5537

# Supplementary materials and methods

Reference numbers refer to the main text list

## SNP and methylation arrays

Copy number profiles were produced using ASCAT (2.5.1) [22]. The segmentation parameter was adjusted to 200 to reduce artefactual segmentation noted on comparison with samples also subjected to copy number analysis from whole genome sequencing.

Methylation profile raw data were quality controlled and pre-processed using the minfi R package (1.30.0) [23]. Probes that were ambiguously mapped or located on the sex chromosomes, contained SNPs, or were not included on both EPIC/450k platforms were filtered out. Raw fluorescence values were corrected for background fluorescence using negative control probes and the minfi background correction function. Dye biases were corrected by scaling all values by a factor required to scale the mean red and green control probes intensities to 10 000. Probe methylation beta values were calculated using the default minfi function in ChAMP [9].

## Methylation data clustering

For unsupervised clustering, a cohort was assembled with all available GCTs, malignant H3.3 tumours, and chondroblastoma (supplementary material, Table S2). Though more osteosarcoma samples were available, a random sample of equivalent number (*n* = 42) to the next largest group, GCT, was used so as not to dominate clustering effects. Unsupervised clustering was performed using the 5000 most variable (by standard deviation) probes across samples. Beta values were transformed to a distance matrix of (1 – correlation values) for hierarchical clustering and dimension reduction analysis. T-distributed stochastic neighbour embedded [t-SNE using the Rtsne package (0.15)] and principal component analysis plots were scrutinised to exclude significant non-biological batch effects introduced by different analysing centres, array platforms or sample types. Hierarchical clustering was then visualised using the packages ape (5.3) and dendextend (1.12.0). Two-dimensional projections were produced using multi-dimensional scaling (MDS) in the MASS package (5.3). Samples were assigned methylation clusters (‘M’, ‘G’, ‘C’, ‘Os’), with names to reflect the predominant disease type within that cluster, by cutting the hierarchical tree at the level of the four principal clades.

## Methylation genomic analysis

Differentially methylated probes and regions were detected using the ChAMP package (2.14.0). Comparison was made between benign and malignant samples with methylation clusters concordant with their diagnoses. Bespoke analysis of genome-wide methylation difference was performed after calculating the signal–noise ratio (SNR) at each probe position. This was calculated as the difference between the mean beta value for each group divided by the pooled variance across the groups. Segmentation was performed using adapted circular binary segmentation (CBS) functions from the DNACopy package (1.58.0). Segmentation was adapted to require a minimum of 10 probes per segment and a segmentation alpha value of 1 × 10^−10^ to prevent spurious oversegmentation. Permutation analysis was conducted by permuting the diagnostic labels randomly and repeating segmentation as above. 10 000 permutations were performed, and the threshold of significant SNR aberration set from the distribution of segment values (<−3 or >3, each *p* < 0.003).

Gene set enrichment analysis (GSEA) was performed using an adapted approach from the ebBayes function in the ChAMP package. In brief, a global test was used to assess the statistical difference between the diagnostic groups across all probes assigned to each gene. This probability value was used as the magnitude of the methylation difference for each gene, while a direction of change was inferred by the mean change in beta value for all gene probes in that gene. GSEA was performed using the fgsea package (1.10.0) across the following gene sets downloaded from the Broad Mutational Signatures Database (MutSigDB) [24]: C2 Reactome, C2 Kegg, C5 Gene Ontology, C6 oncogenic pathways, C7 immunology pathways and the hallmarks pathways.

## Methylation-based copy number analysis

Methylation array-based copy number analysis was performed using a binned intensity track generated using the *conumee* package (1.18.0). Raw data for 119 control male and female diploid samples were downloaded from GEO (GSE109381). Binned intensity values were fitted to integer copy number states using the principles underlying the ASCAT package [22]. In brief, a grid of possible purity and ploidy values was searched to minimise the sum of Euclidean distances between the intensity-based inferred number of copies and integer values.

## Image cytometry

## Based on a previously reported but modified protocol [25], 50-µm-thick formalin-fixed and paraffin-embedded tumour sections were deparaffinised and rehydrated. Nuclear suspensions were obtained through cytoplasmic digestion using protease type VIII (P5380; Sigma-Aldrich, Gillingham, UK). Samples were filtered, cytospun, and subjected to DNA hydrolysis (5 m HCl) and Feulgen staining (Schiff’s fuchsin-sulphite reagent; S5133, Sigma-Aldrich). DNA ploidy was measured using the Fairfield DNA ploidy system (Fairfield Imaging, Kent, UK). A histogram with a DNA index was produced for each sample by calculating the integrated optical density.

## Variant detection and validation

The precision of *Cancer Genome Project* (Wellcome Trust Sanger Institute) variant calling pipeline has been determined in multiple studies and utilised the following algorithms [26]:

| Function | Name | Version | Reference |
| --- | --- | --- | --- |
| WGS alignment | Burrows–Wheeler Aligner (BWA mem) | 2.0.54 | [27] |
| Substitutions | Cancer Variants through Expectation Maximisation (CaVEMan) | 1.11.0 | [28] |
| Indels | cgpPindel | 2.2.4 | [29] |
| Copy number | Battenberg (with SVs from BRASS) | 2.2.8 | [12,30] |
| Structural variants | Breakpoints via Assembly (BRASS) | 5.3.2 | <https://github.com/>  cancerit/BRASS |
| Telomere lengths | TelSeq | 0.0.1 | [31] |

This was confirmed through manual inspection of raw sequencing reads for up to 100 variants of each type from all samples. The precision of all variants was >95% in all cases. Additional post-processing filters were applied to substitutions to achieve this precision: median alignment score (ASMD) of variant reads >90 (100 bp paired end) and >140 (150 bp paired end) and median number of clipped bases in variant supporting reads (CLPM) of 0. Sample PD37332 was noted to have a large number of structural variants supported by minimal numbers of locally discordantly mapping reads; therefore, for this sample only, structural variants were only considered if they were possible to map to base-pair resolution. All copy number profiles were manually scrutinised for the requirement of refitting; however, in all cases, the first solution was optimal.

## Mutation clustering, purity estimation, and phylogenetic reconstruction

The algorithm DPClust (2.2.6) and its pre-processing pipeline (1.0.8) were used to cluster mutations according to fraction of cancer cells (cancer cell fraction, CCF) in which they were found, as described previously [12]. Filtered substitutions and Battenberg copy number profiles were used as input. For samples with significant copy number aberrations, purity estimates derived from Battenberg were considered accurate. For samples without significant copy number aberration, purity was estimated with an initial run of DPClust on only balanced segments (1+1), to derive the VAF of the clonal cluster, which reflects the 2/purity. This purity value was then used for a definitive run of DPClust. All individual samples were run with DPClust in single-sample mode, while PD38329 was also run as a multi-sample case.

Three-dimensional clustering plots were produced for PD38329 with the R packages rgl (0.1) and htmlwidgets (1.3). Phylogenetic reconstruction was performed using the pigeon-hole principle as previously described [12]. In brief, subclones were designated to be nested within a clone or another subclone if their combined CCF exceeded that of their parent.

## Simple timing analysis

Initial timing analysis required the transformation of individual mutation allele frequencies into mutation copy number. This was performed using the equation:

$$MCN =\frac{VAF(\rho\times TCN+2\left( 1-\rho\right))}{\rho}$$

where *MCN* is the mutation copy number, *ρ* is the sample purity, and *TCN* is the local total copy number.

For whole genome duplication (WGD) timing, deamination (clock-like, C>T mutations at CpG dinucelotides) mutations were selected from regions of balanced gain (2+2) or LOH (2+0). A probabilistic approach to WGD timing was taken. Each mutation had a probability assigned for being at each mutation copy number state up to and including the major allele copy number state at that locus. This probability was calculated from a binomial distribution based on the sequencing depth and the measured allele frequency. An estimate for WGD was estimated as:

$$WGD =\sum\frac{p{Mut}_{2}}{{pMut}_{2}+ \frac{{pMut}_{1}}{2}}$$

where ${pMut}_{x}$ is the probability of a mutation being at a copy number *x*. ${pMut}_{1}$ is divided by *x*_max_ (in this example, 2) to account for the increased genomic material after genomic duplication. The fractional estimate of WGD is then scaled to the patient’s age at diagnosis for a real-time estimate of WGD. Confidence intervals are created by 1000 bootstrap iterations, resampling the underlying mutations. In PD4922e, regions with major allele copy number of 3 in WGD samples were assumed, by parsimony, to have been acquired by a copy number gain subsequent to WGD. This is because the only alternative explanation would be a deletion after a second local duplication and since only a single genome duplication occurred, this was considered unlikely. The timing of this additional gain was computed by calculating the proportion of clock-like mutations (again probabilistically) that were acquired prior to WGD (at MCN 3), between WGD and additional copy number gain (MCN 2) and after this gain (MCN 1).
