# Supplementary material for: Drivers underpinning the malignant transformation of giant cell tumour of bone
Source: J Pathol. 2020 Oct 6;252(4):433–40. doi: 10.1002/path.5537 (PMC8432151; doi:10.1002/path.5537)

Drivers underpinning the malignant transformation of giant cell tumour of bone
MW Fittall *et al.* *J Pathol* DOI: 10.1002/path.5537

Figure S1. Telomere length (tumour minus normal telomere length with error bars reflecting the 5th and 95th centiles from 10,000 bootstrapped estimates sampling the data from the underlying sequencing read groups), mutation burden, structural variant (SV) burden and copy number (CN) score for sequenced tumours. Tumours are ordered by their burden of SNVs.

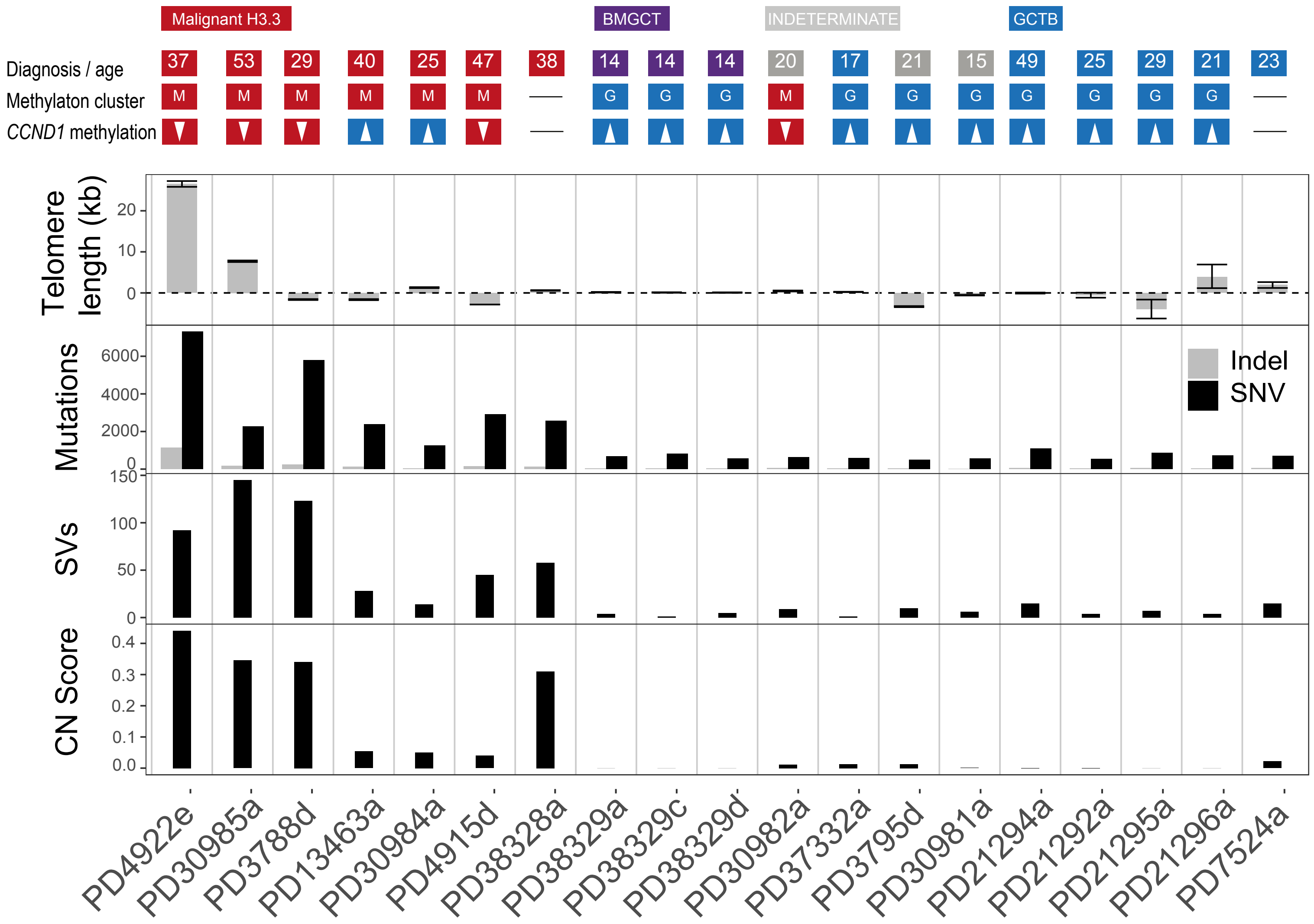

Figure S2. *TERT* rearrangement in PD3788d. Copy number is shown with the orange (total copy number) and black (minor allele) segments counted from a minimum of zero on the innermost ring. Structural variants are shown as coloured internal links (blue – tandem duplications, red – deletions, yellow – inversions, grey – translocations. Genes lying within 10kb of each breakpoint are labelled on the outside. The random configuration of rearrangements and deletions, typical for chromothripsis is seen. Of note, this sample has undergone whole genome duplication.

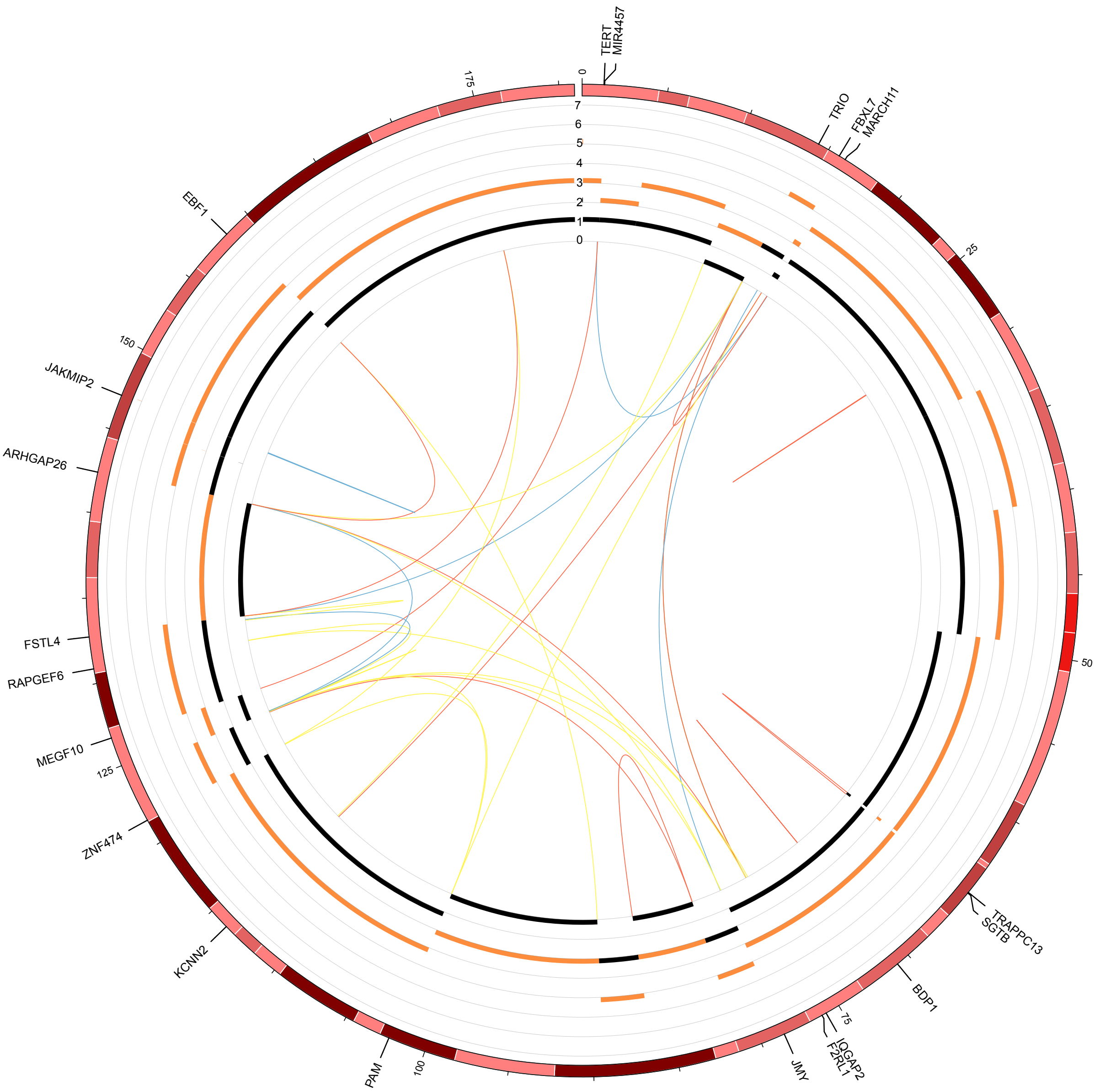

Figure S3. KDM4B homozygous deletion and RB1 loss of heterozygosity. (A) PD38328a KDM4B homozygous deletion. The different transcripts for KDM4B are shown at the top. LogR represents logged normalised ratios of tumour and normal coverage at SNP positions. The red link is an annotation of a deletion that was orthogonally detected by the structural variant caller BRASS. The loss of heterozygosity of 19p is not visible at this scale, therefore B-allele frequencies are not shown. (B,C) Loss of heterozygousity on chromosome 13 (including *RB1*) is shown in for the samples with Alternative Lengthening of Telomeres, (B) PD4922e and (C) PD30985a.

A PD38328a

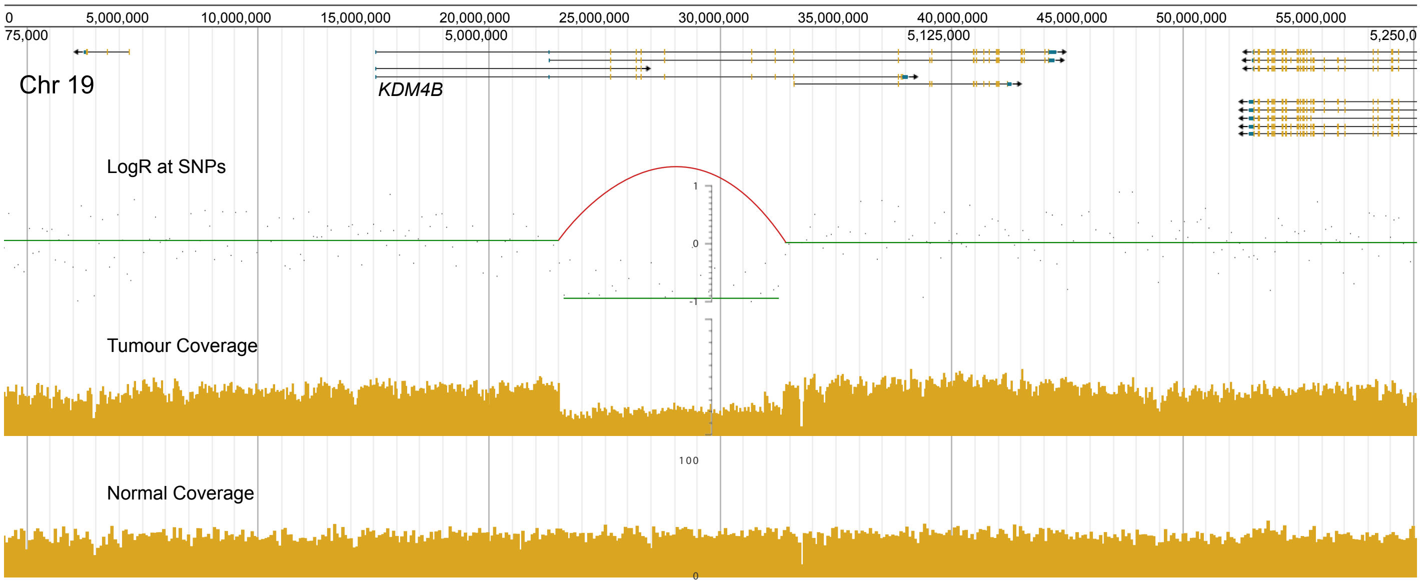

B

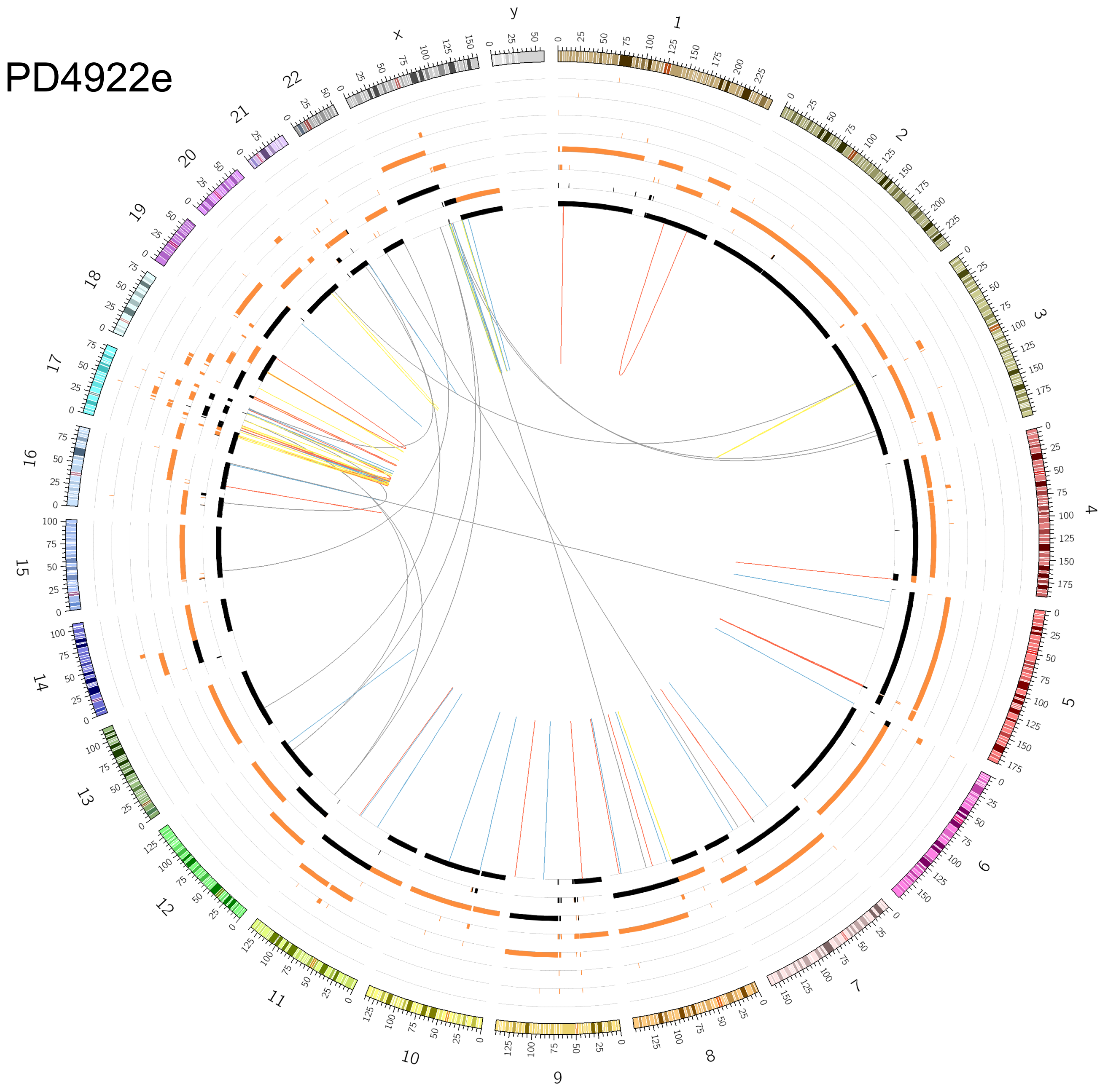

C

PD30985a

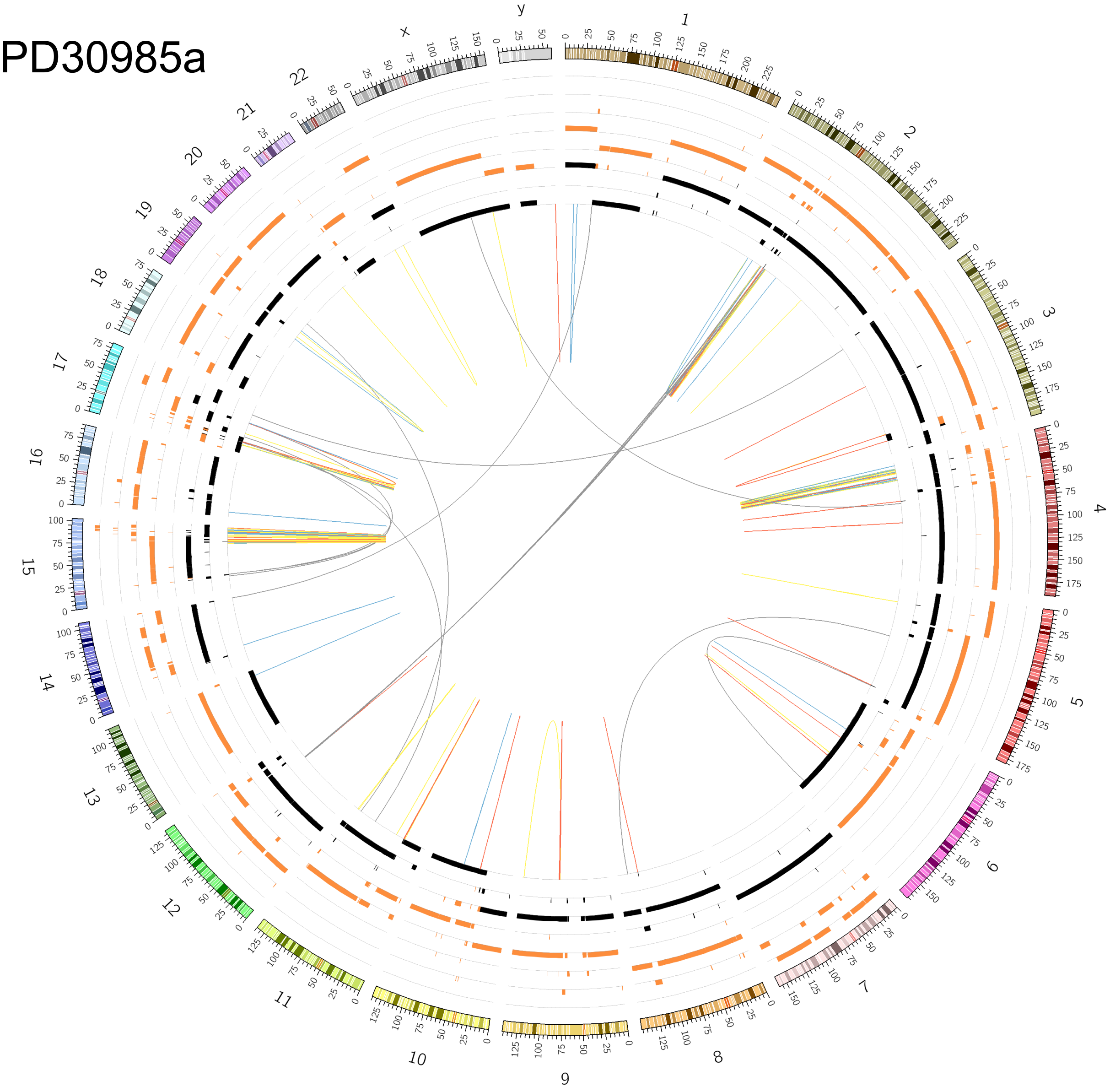

Figure S4. SNP and Methylation array-based copy number scores. Array-based copy number scores (0-1, representing the theoretical maximal range of aneuploidy). (A) SNP array-based copy number scores. (B) Methylation array-based scores.

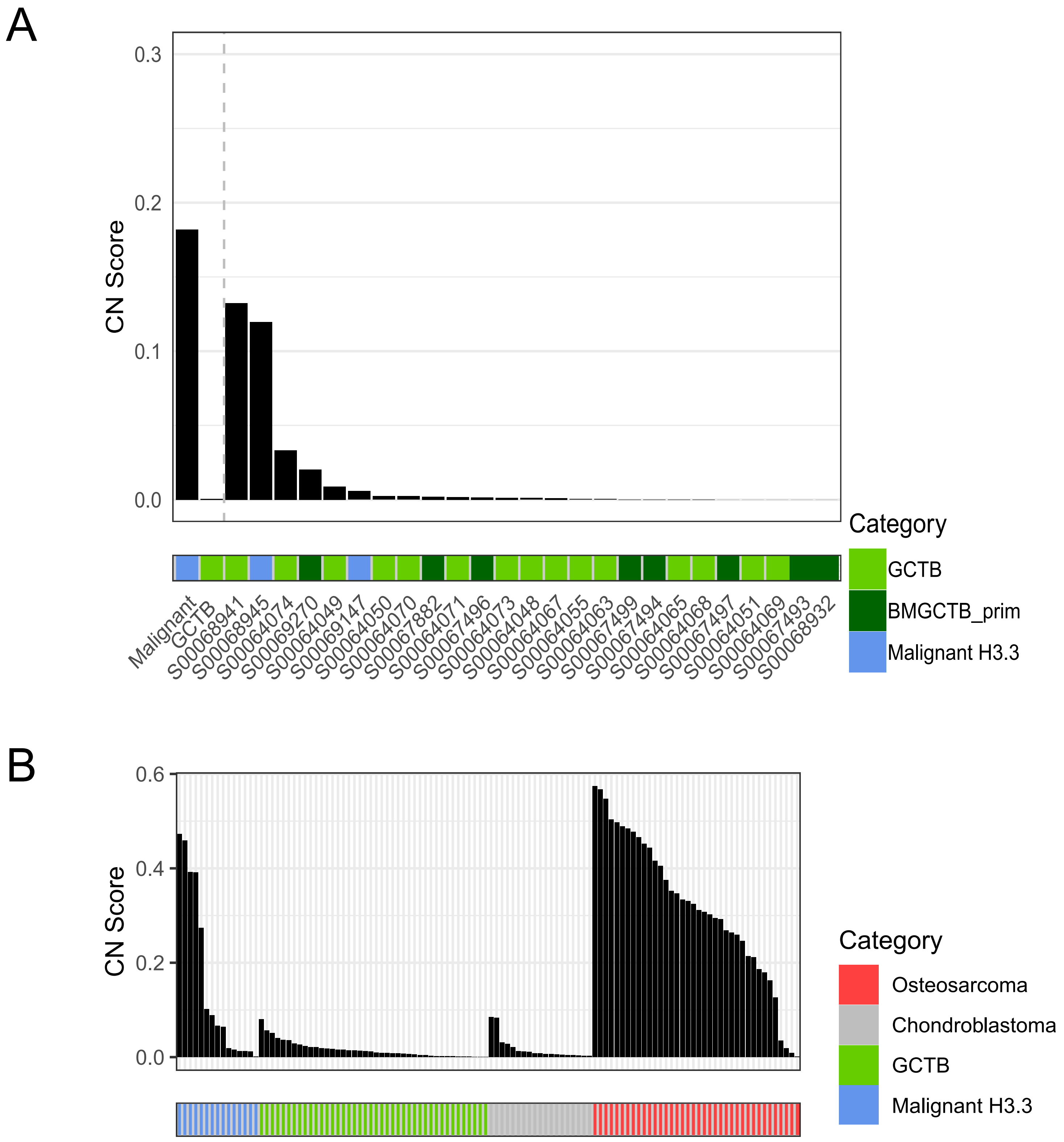

Figure S5. Mutation timing of malignant samples. For each sample, PD30985a, PD4922e and PD3788d shown as indicated: (A) Copy number plot for chromosome containing histone mutations (orange segments - total copy number, black segments – minor allelic copy number). Each point is a mutation allele frequency transformed into mutation copy number. Red dots are driver mutations. (B) Timeline of driver mutation acquisition and genome duplication. Genome duplication is marked in the grey bar with a confidence interval generated by 10,000 bootstrapped iterations resampling mutations. The red line, in sample PD4922e, which was the only one with sufficient additional gains to commute, is the real-time timing for additional gains (>3 major allele) with a confidence interval in yellow. (C) Complex rearrangement events resembling chromothripsis.[4]

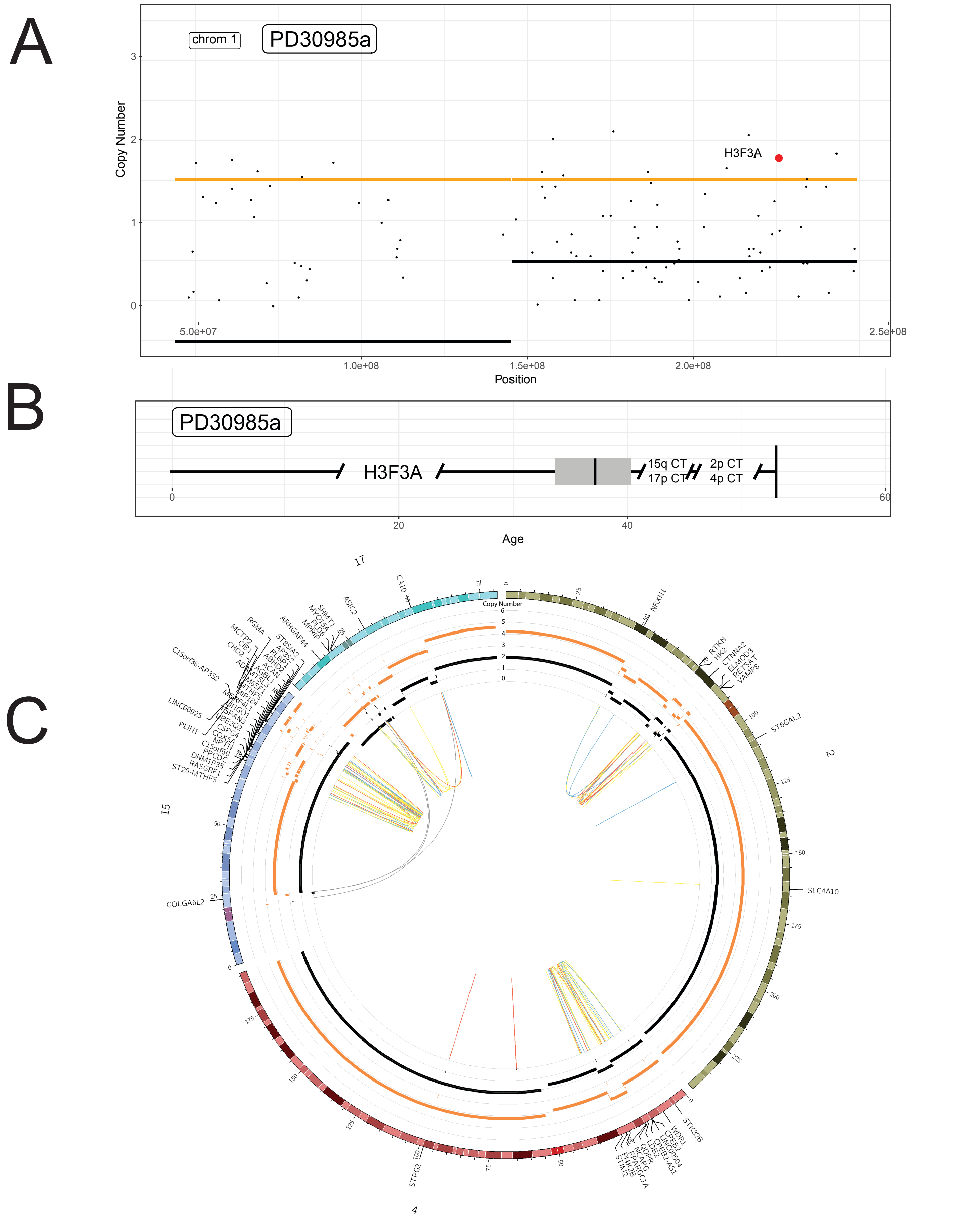

A

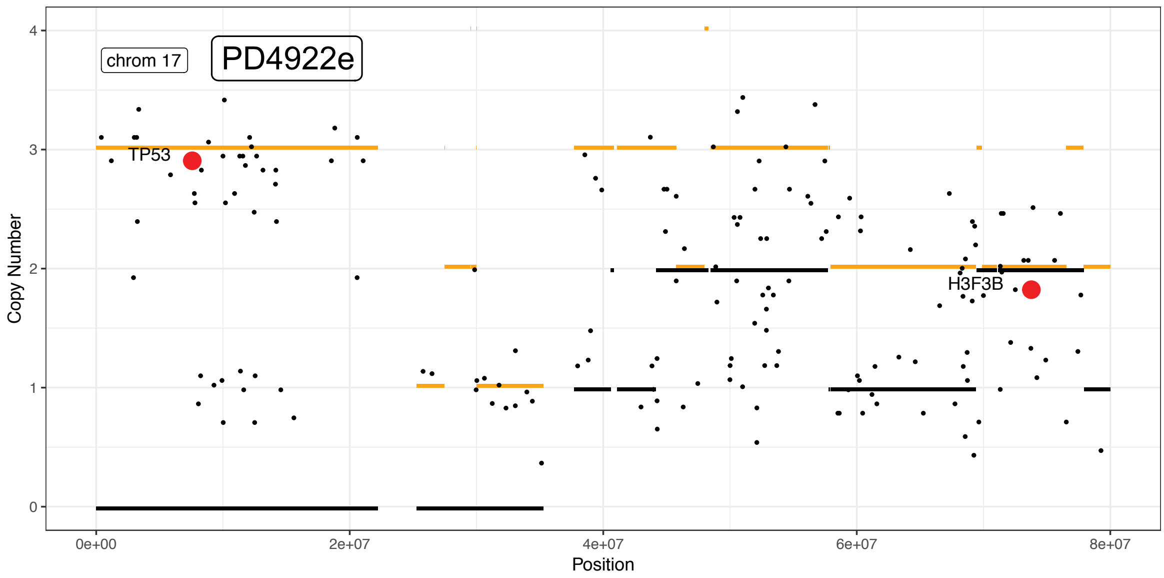
chrom 17
PD4922e
TP53
H3F3B
Copy Number
Position
0e+00
2e+07
4e+07
6e+07
8e+07

B

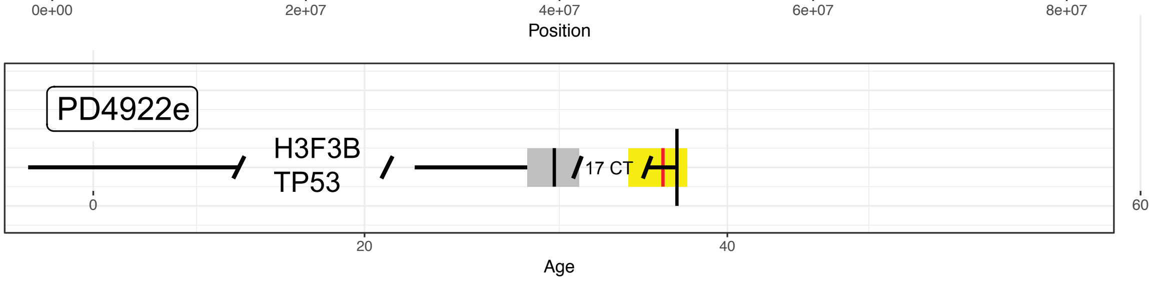
PD4922e
H3F3B
TP53
17 CT
Age
0
20
40
60

C

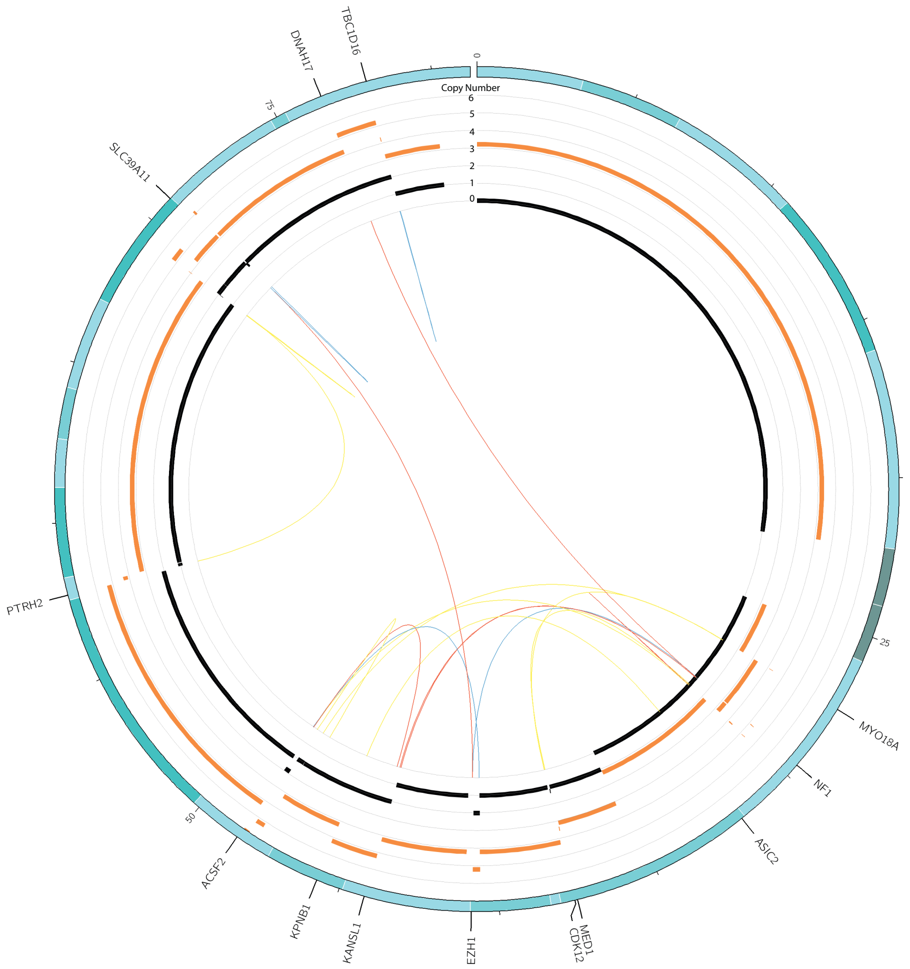
Copy Number
TBC1D16
DNAH17
SLC39A11
PTRH2
ACSF2
KPNB1
KANSL1
EZH1
MED1
CDK12
ASIC2
NF1
MYO18A

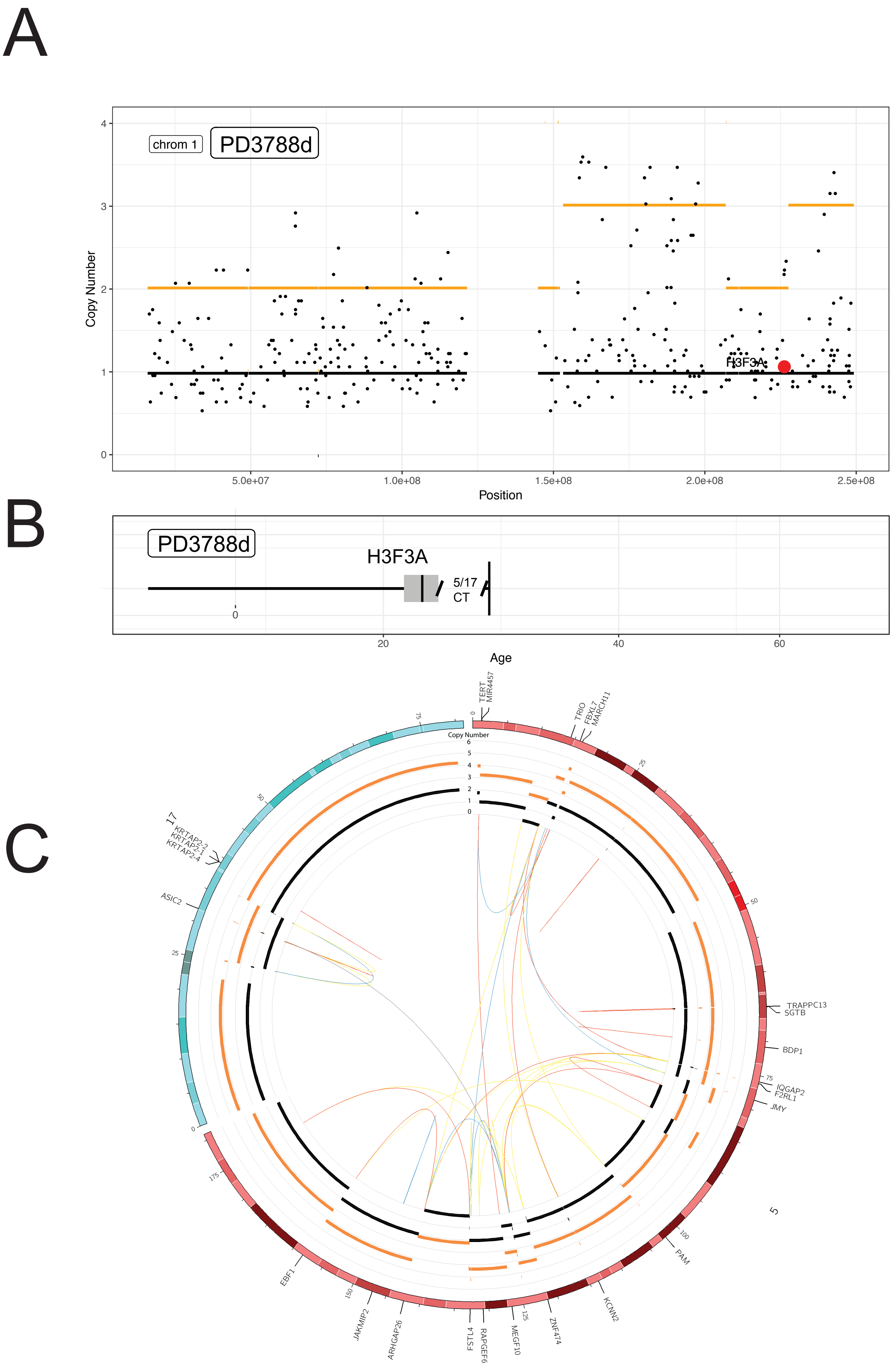
A
chrom 1
PD3788d
Copy Number
Position
5.0e+07
1.0e+08
1.5e+08
2.0e+08
2.5e+08
H3F3A
B
PD3788d
H3F3A
5/17
CT
Age
0
20
40
60
C
Copy Number
TERT
MIR4457
TRIO
FBXL7
MARCH11
TRAPPC13
SGTB
BDP1
IQGAP2
F2RL1
JMY
PAM
KCNN2
ZNF474
MEGF10
RAPGEF6
FSTL4
ARHGAP26
JAKMIP2
EBF1
ASIC2
KRTAP2-2
KRTAP2-1
KRTAP2-4
17
5

Figure S6. Mutation clustering in metastatic samples. Mutation clustering demonstrating the pattern of metastatic seeding in benign-metastasising GCT. (A) Two-dimensional projections of clustering of mutations by their CCF. Each dimension is one sample, points represent mutations. Points are coloured by the cluster to which they were assigned by DPClust. Mutations clustering at CCF=1 (green) are clonal in all samples and therefore define mutations found in the most recent common ancestor to all sampled cells. (B) clonal phylogenetic trees defined by mutation clustering for each sample. The length of connecting lines denotes the number of mutations acquired, the area of each circle is proportional to the number of cells possessing those mutations. The position of each subclone is defined by the ‘pigeon-hole’ principle: if the sum of the CCFs of two subclones is greater than their mutual parent, then one must lie within the other. (C) The pattern of clonal spread from primary tumour to metastases.

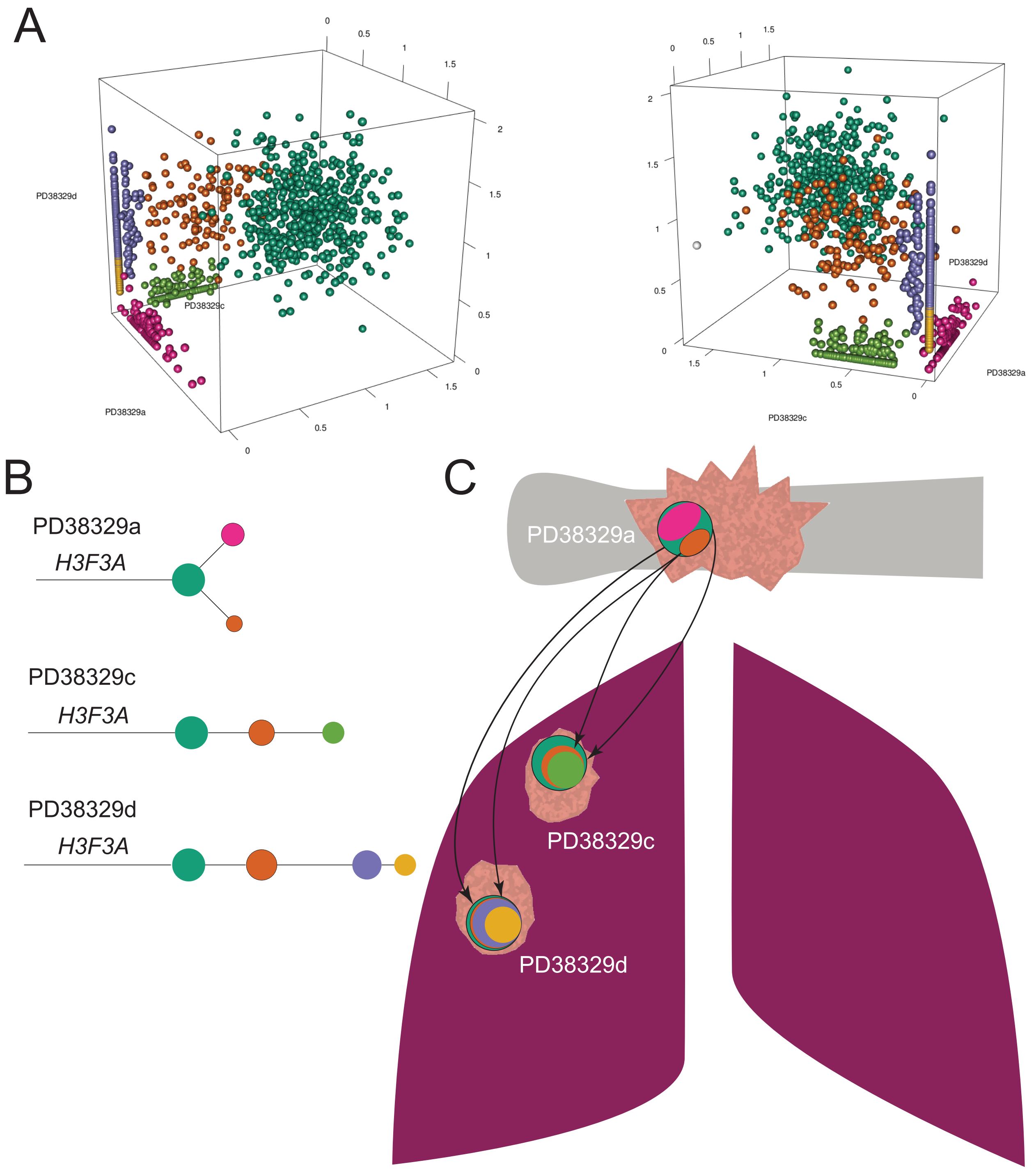

Figure S7. Tumour methylation clustering with other malignant bone tumours. Methylation clustering (as per Figure 2) with 19 adamantinomas and 36 pleomorphic sarcomas. Histone-mutated tumour clades (GCTB, malignant H3.3 tumours and chondroblastoma) are robust and distinct from these tumour entities; pleomorphic sarcomas also seem predominantly to form their own clade. Adamantinomas are also segregated, with some similarity to one osteosarcoma clade.

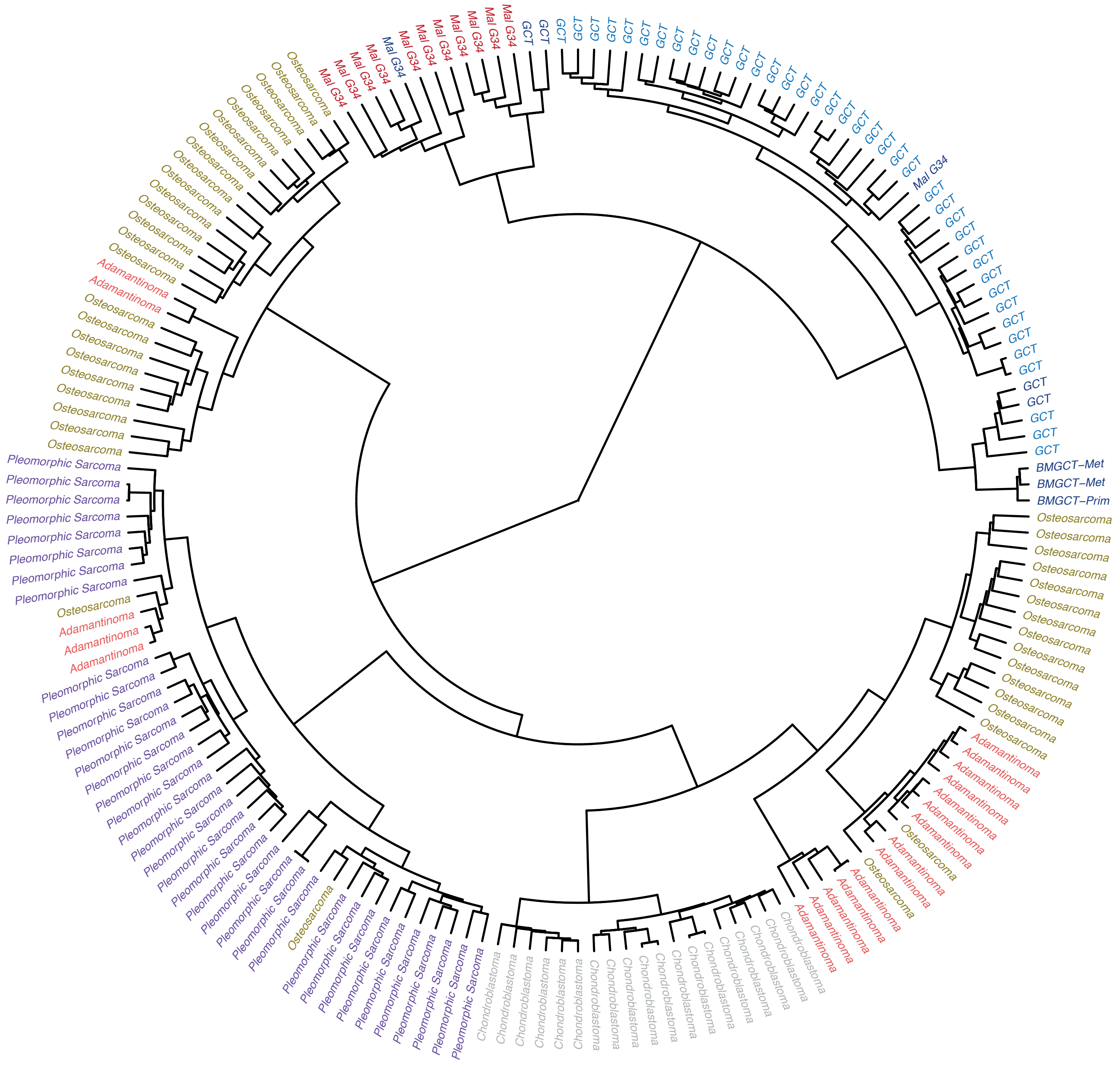

Figure S8. Selected gene promoters identified as differentially-methylated regions. Focal differentially-methylated regions (DMRs). Representative examples of the 56 DMRs focally identified to target gene transcription start sites. Grey points are aggregated signal-noise ratios (SNR) of malignant to benign methylation group differences. Green segments are segmented SNR values. Genes are denoted with a grey bar with a black tick identifying the known transcription start site whilst the blue segment is the ChAMP identified DMR.

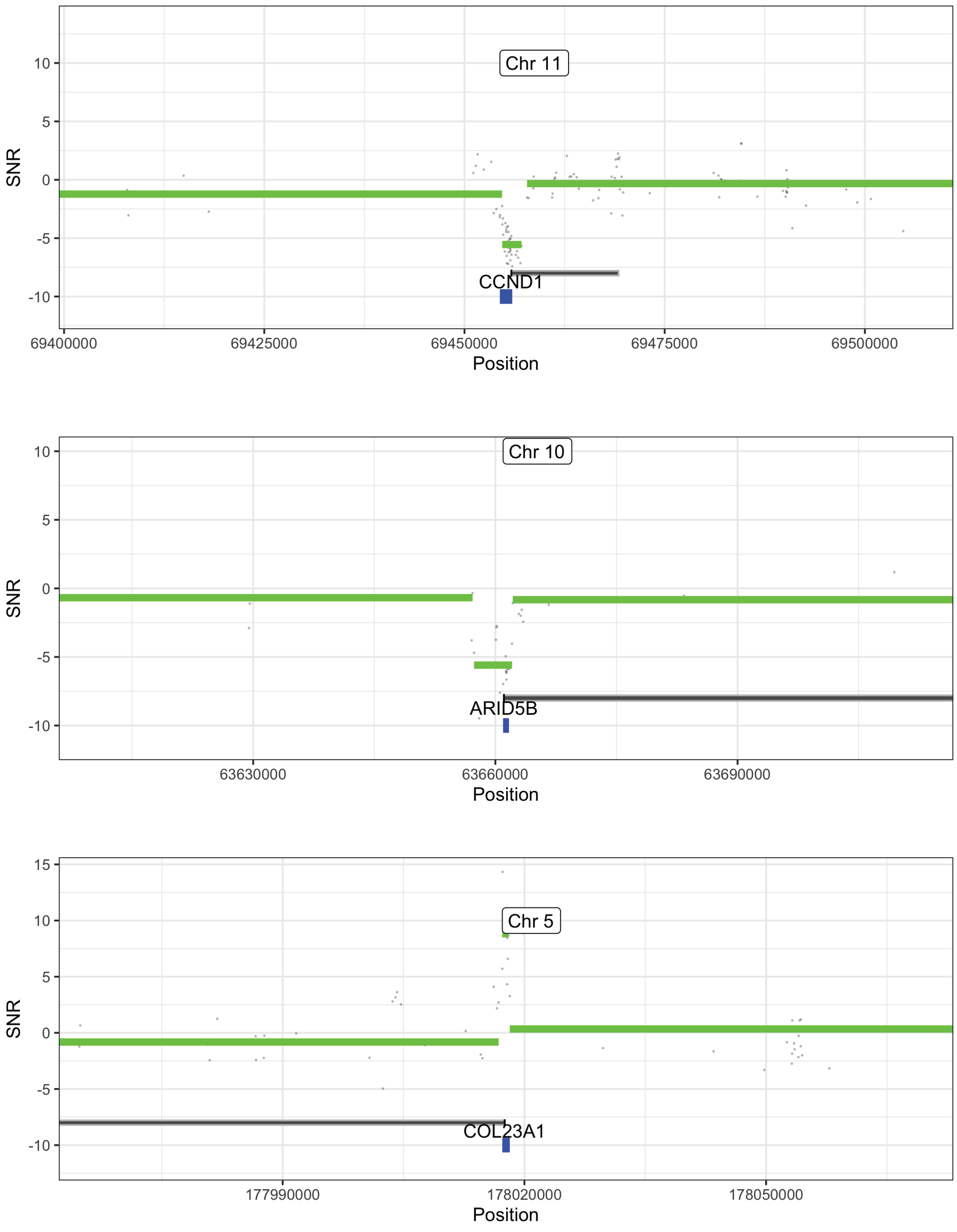

Figure S9. Whole genome bisulphite sequencing confirmation of *CCND1* differential methylation. The proportion of sequencing reads supporting cytosine methylation are shown for all CpG sites across *CCND1* for the available tumours.

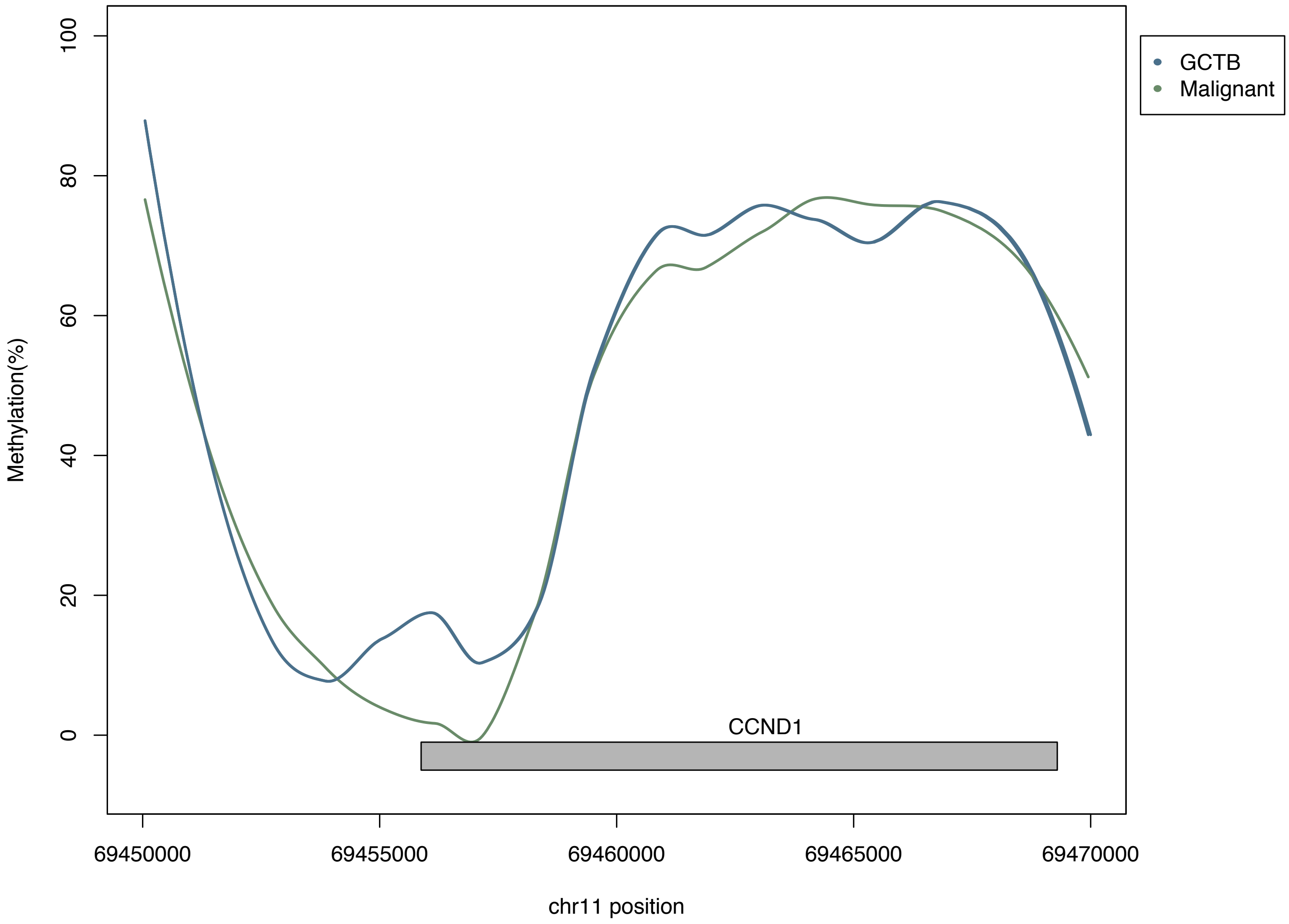

Figure S10. Differential methylation in the HIST1 cluster. The upper panel displays the signal-noise ratio (SNR) of differential methylation between malignant H3.3-mutated tumours and GCTB, with differentially methylated regions annotated at blue ticks in the panel. The lower panel highlights a region of the HIST1 cluster of histone genes which are consistently hypermethylated in malignant H3.3 tumours.

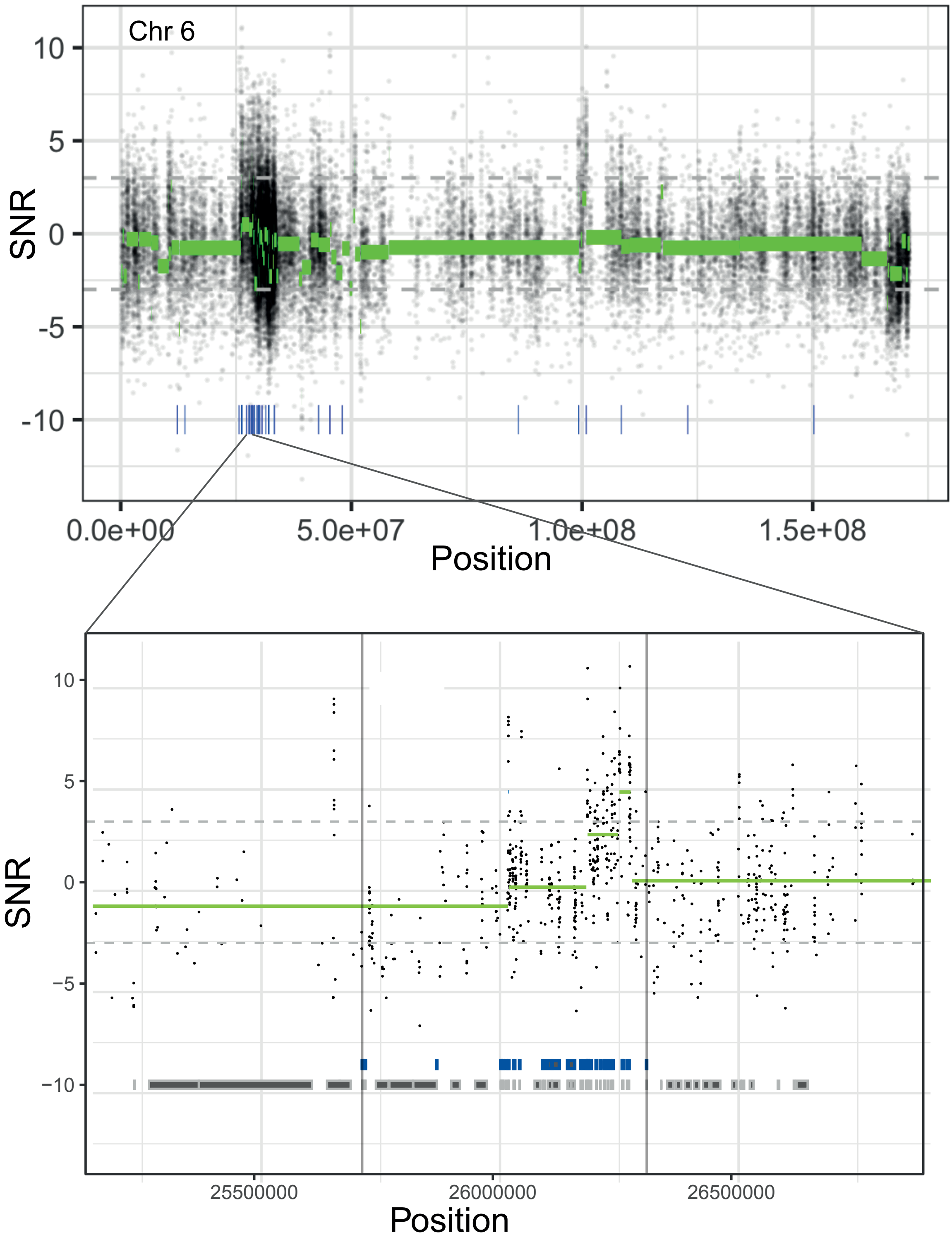

Figure S11. The loss of *H3-3* mutation in the malignant GCTB sample PD38328a. (a) Haematoxylin and Eosin (H&E)-stained tissue section of a tumour biopsy showing an atypical spindle cell sarcoma without osteoclasts with focal immunoreactivity for the H3.3 G34W-mutant protein. (B) H&E of the same spindle cell tumour at resection showing complete loss of H3.3 G34W immunoreactivity. (C) Circos plot of rearrangements (internal links) and copy number (total in orange, minor allele in black) demonstrating loss of heterozygosity at 1q (*H3-3A* locus).

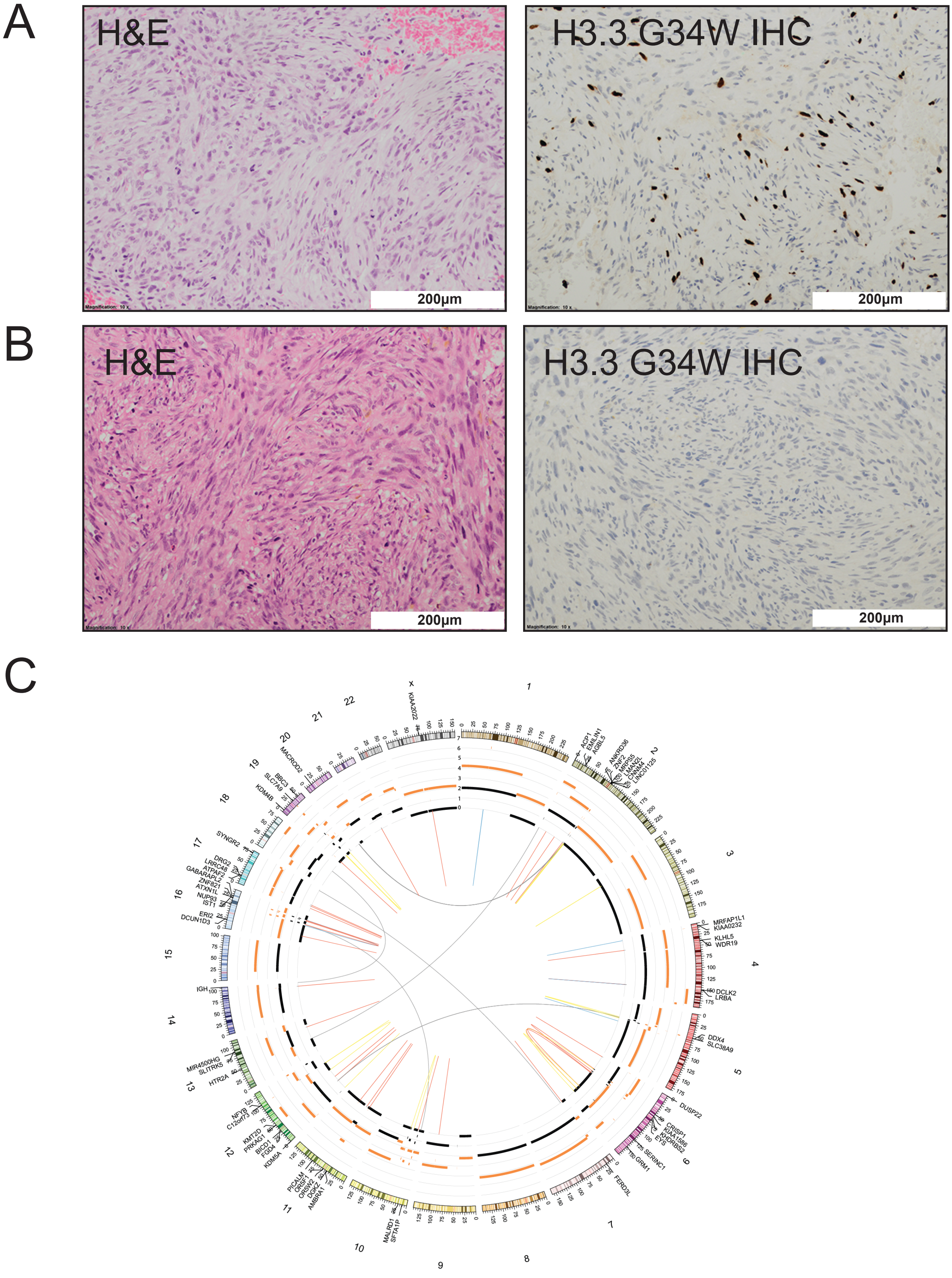

Supplement: Supplementary file 2 — Figure S1. Telomere lengths, mutation burdens, structural variant burdens, and copy number scores for sequenced tumours Figure S2.TERT rearrangement in PD3788d Figure S3.KDM4B homozygous deletion and RB1 loss of heterozygosity Figure S4. SNP and methylation array‐based copy number scores Figure S5. Mutation timing of malignant samples Figure S6. Mutation clustering in metastatic samples Figure S7. Tumour methylation clustering with other malignant bone tumours Figure S8. Selected gene promoters identified as differentially‐methylated regions Figure S9. Whole genome bisulphite sequencing confirmation of CCND1 differential methylation Figure S10. Differential methylation in the HIST1 cluster Figure S11. The loss of H3‐3 mutation in the malignant GCTB sample PD38328a [file PATH-252-433-s002.pdf]
