# Supplementary material for: Drivers underpinning the malignant transformation of giant cell tumour of bone
Source: J Pathol. 2020 Oct 6;252(4):433–40. doi: 10.1002/path.5537 (PMC8432151; doi:10.1002/path.5537)
Supplement: Supplementary file 4 — Table S3. Clinically indeterminate cases [file PATH-252-433-s001.docx]

**Drivers underpinning the malignant transformation of giant cell tumour of bone**

MW Fittall *et al. J Pathol* DOI: 10.1002/path.5537

**Table S3.** Clinically indeterminate cases

| **Identifier** | **Clinical/histological findings (analysed specimen in bold)** | **Genetic findings** | **Methylation findings** | **Comment** |
| --- | --- | --- | --- | --- |
| PD30981a | **Aged 15 right tibial GCTB resected**.  Aged 17 local recurrence resulting in re-resection: Osteoclast-rich lesion with indeterminate appearances (fibroblastic overgrowth with malignant osteoid deposition, mitotically active spindle cells with atypical mitotic figures), reviewed in two national specialist bone pathology units. Initial and recurrent histology diagnosis revised to high-grade osteosarcoma. Adjuvant chemotherapy given, with no evidence of recurrence in 13 years of follow-up.  Current review of histology would suggest the findings are consistent with an atypical but benign GCTB | No additional drivers, low CN score | GCTB cluster, hyper-methylated *CCND1* | Genetic and epigenetic findings in the primary lesion supported the original benign diagnosis |
| PD30982a | **Aged 20 right proximal fibular tumour which underwent resection.** Spindle cell tumour with diffuse osteoid deposition and scattered osteoclasts. Numerous mitoses amongst spindle cells which are large and have a moderate degree of nuclear polymorphism. Reviewed in three specialist bone tumour units with diagnoses ranging from benign GCTB, through low- and high-grade osteosarcoma. Managed as a high-grade osteosarcoma, therefore adjuvant chemotherapy given with no evidence of recurrence in 13 years of follow-up | No additional drivers and low CN score | Malignant H3.3 cluster, hypo-methylated *CCND1* | Epigenetic findings support the clinical diagnosis of a malignant H3.3-mutated tumour though without additional driver mutations and aneuploidy |
| PD3795d | Aged 21 left 1st metatarsal conventional GCTB treated with curettage.  **Age 29 local recurrence – biopsy suggested GCTB, therefore proceeded to curettage –** primary report suggested GCTB though noted frequent mitoses, areas of necrosis, and local cortical bone erosion. Sent for a second opinion which suggested a diagnosis of osteosarcoma. Chemotherapy therefore given before a 1st ray amputation. Amputation specimen, 1st ray amputation – extensive necrosis with fibrocystic change with no residual viable tumour (presumed excellent chemotherapy response). No recurrence in 12 years of follow-up | No additional drivers, low CN score | GCTB cluster, hyper-methylated *CCND1* | Epigenetic and genetic findings would not have supported the diagnosis of malignant transformation which led to the use of chemotherapy |

| PD38329a/c/d | **Aged 14 spontaneous distal left radial fracture. Biopsy suggested GCTB, therefore proceeded to resection.**  Six months later developed chest pain and imaging revealed bilateral pulmonary metastases. **Lung lesion biopsy** suggested a giant-cell rich osteosarcoma. Proceeded to two cycles MAP chemotherapy with no clinical response. Secondary histological review suggested the primary and metastatic lesions are all consistent with conventional GCTB | No additional drivers, low CN score | GCTB cluster, hyper-methylated *CCND1* | Epigenetic and genetic findings support a diagnosis of metastatic benign GCTB |
| --- | --- | --- | --- | --- |
| 100994770004_R06C01 | **Aged 20 presented with a sacral lesion.** Biopsy suggested an osteoclast-rich lesion and therefore the patient proceeded to curettage. Focal mild stromal cell atypia within an osteoclast-rich tumour made the differentiation between a low-grade osteosarcoma and giant cell tumour of bone challenging. The tumour was reviewed by a number of specialist bone pathologists internationally, with the balance of opinion supporting a diagnosis of giant cell tumour of bone. No recurrence in 3.5 years of follow-up |  | GCTB cluster, hyper-methylated *CCND1* | Epigenetic findings would support the diagnosis of GCTB |
| 100994770004_R06C01 | **Aged 18 presented with a 7th left rib lesion which underwent resection.** Histologically, this represented an unusual bone-forming, osteoclast-rich lesion which was difficult to classify with only mild atypia. Differential diagnosis lay between a low-grade osteosarcoma and GCTB. After multiple international pathology reviews, the balance of opinion supported GCTB. No recurrence after 3 years of follow-up |  | Malignant methylation profile but CN score of 0.014 (in keeping with GCT) | The epigenetic profile supports the initial suggestion of malignant transformation |

| 100994770004_R04C02 | Aged 19, presented with a tibial lesion initially reported as atypical fibrous histiocytoma (AFH).  Aged 23 developed multiple spinal lesions; biopsy was reported as consistent with the initial tibial lesion, AFH. Underwent adjuvant chemotherapy (ifosfamide, then doxorubicin and cisplatin), then radiotherapy (46 Gy in 24 fractions).  Aged 30 developed a lesion in the odontoid peg (C2) which underwent resection and adjuvant radiotherapy (46 Gy in 23 fractions). This and all previous lesions reviewed in multiple specialist pathology units and suggested that they were all consistent with osteoclast-rich tumours without evidence of malignancy, therefore multi-focal GCTB.  **Aged 35 developed a T2/3 paravertebral lesion which underwent resection and radical cyberknife radiotherapy (24 Gy in 3 fractions).** Abnormal mitotic figures were present and suggested malignancy and contemporaneous diagnosis of osteoclast-rich osteosarcoma, consistent with secondary malignant transformed GCTB.  Aged 43 developed a chest wall lesion, histology again consistent with a high-grade osteoclast-rich osteosarcoma – secondary malignant GCTB. Deceased as a consequence of previous surgical prosthetic erosion into the aorta and oesophagus, prior to further therapy |  | GCTB cluster and hypermethylated *CCND1* but CN score of 0.1 (from methylation array) is markedly outside of distribution for GCT | The epigenetic changes reflect the initial GCTB-like findings; the degree of aneuploidy is not concordant with this and the clinical behaviour, and histological transformation may therefore reflect the impact of chemotherapy and radiotherapy |
| --- | --- | --- | --- | --- |
